# Comprehensive evaluation of AlphaFold/OpenFold prediction of experimentally unresolved proteins through novel metrics

**DOI:** 10.1093/bib/bbag461

**Published:** 2026-09-03

**Authors:** Florencia R Díaz, Daniela Orschanski, Juan I Folco, Juana Espain Ceci, Guadalupe Nibeyro, Horderlin Robles Vega, Juan P Nicola, Elmer A Fernández

**Affiliations:** Facultad de Ciencias Exactas Físicas y Naturales (FCEFyN), Universidad Nacional de Córdoba, Av. Vélez Sarsfield 1611, Ciudad Universitaria, X5016GCA, Córdoba, Argentina; ScireLab, Fundación para el Progreso de la Medicina, 9 de Julio 941, Centro, X5000EMS, Córdoba, Argentina; ScireLab, Fundación para el Progreso de la Medicina, 9 de Julio 941, Centro, X5000EMS, Córdoba, Argentina; Consejo Nacional de Investigaciones Científicas y Técnicas (CONICET), Córdoba, Argentina; Facultad de Ingeniería, Universidad Católica de Córdoba, Av. Armada Argentina 3555, Campus Universitario, X5016DHK, Córdoba, Argentina; Facultad de Ciencias Exactas Físicas y Naturales (FCEFyN), Universidad Nacional de Córdoba, Av. Vélez Sarsfield 1611, Ciudad Universitaria, X5016GCA, Córdoba, Argentina; ScireLab, Fundación para el Progreso de la Medicina, 9 de Julio 941, Centro, X5000EMS, Córdoba, Argentina; Consejo Nacional de Investigaciones Científicas y Técnicas (CONICET), Córdoba, Argentina; Grupo GNOCIX, Facultad de Ciencias e Ingenierías, Universidad del Sinú Elías Bechara Zainum, Cra. 1W No. 38-153, Barrio Juan XXIII, 230001, Montería, Córdoba, Colombia; Departamento de Bioquímica Clínica, Facultad de Ciencias Químicas, Universidad Nacional de Córdoba, ica, Facultad de Ciencias Químicas, Universidad Nacional de Córdoba, Haya de la Torre y Medina Allende s/n, Ciudad Universitaria, X5000HUA, Córdoba, Argentina; Centro de Investigaciones en Bioquímica Clínica e Inmunología, Consejo Nacional de Investigaciones Científicas y Técnicas (CIBICI-CONICET), Haya de la Torre y Medina Allende s/n, Ciudad Universitaria, X5000HUA, Córdoba, Argentina; Facultad de Ciencias Exactas Físicas y Naturales (FCEFyN), Universidad Nacional de Córdoba, Av. Vélez Sarsfield 1611, Ciudad Universitaria, X5016GCA, Córdoba, Argentina; ScireLab, Fundación para el Progreso de la Medicina, 9 de Julio 941, Centro, X5000EMS, Córdoba, Argentina; Consejo Nacional de Investigaciones Científicas y Técnicas (CONICET), Córdoba, Argentina; Instituto de Tecnología (INTEC), Universidad Argentina de la Empresa (UADE), Lima 775, Monserrat, C1073AAO, Buenos Aires, Argentina

**Keywords:** protein structure prediction, AlphaFold3, OpenFold, ColabFold, single amino acid variants, intrinsically disordered regions

## Abstract

Predicting accurate protein structures is essential for understanding molecular mechanisms, interpreting the impact of sequence variation, and supporting translational applications ranging from drug discovery to clinical genomics. Recent advances in deep-learning–based predictors such as AlphaFold2, OpenFold, and AlphaFold3 have transformed structural biology, enabling routine in silico modeling even for challenging or previously uncharacterized proteins. However, systematic benchmarking of these tools—especially for novel targets and single amino acid variants—remains limited. Conventional global metrics often fail to capture biologically meaningful discrepancies. By evaluating multiple implementations of AlphaFold2 and OpenFold, together with ColabFold and the AlphaFold3 server, across 10 different proteins and 222 single amino acid protein variants encompassing a wide range of sizes, structures, and functions, we show that although widely used global indicators—like mean pLDDT, pTM-score, and RMSD—frequently suggest comparable performance, substantial local-level differences remain elusive. To address this gap, we introduce a comparative framework leveraging Bland–Altman agreement analysis, to evaluate per-residue Cα-confidence differences and Per-Residue profiles (PRPs), complemented by Uniform Manifold Approximation and Projection (UMAP). This approach reveals marked localized divergences, particularly within flexible or intrinsically disordered regions, where both predictor choice and single-residue substitutions trigger the largest conformational shifts. We further demonstrate that using reduced homology databases has minimal impact on predicted structural quality, offering computationally efficient alternatives. Collectively, our findings underscore the importance of integrating global and residue-specific evaluations to more accurately assess robustness, agreement, and practical usability across contemporary protein structure prediction methods.

## Introduction

Predicting the three-dimensional (3D) structure of proteins from their amino acid sequences has been one of the central challenges in structural biology for decades [1–5]. This task becomes particularly critical for experimentally unresolved proteins—those lacking experimental structural references—whose structures are either unknown or too costly to determine using traditional methods such as X-ray crystallography or cryo-EM [6]. Predicting these unresolved structures is essential to advance our understanding of protein function, explore new therapeutic targets, and enable downstream applications such as molecular docking, protein engineering, and variant effect prediction [7].

Recent advances in deep learning, notably AlphaFold2 (AF2) and AlphaFold3 (AF3) [1, 3], have transformed this field by providing highly accurate predictions for a vast number of protein structures [2, 4, 5]. These models, trained on multiple sequence alignments (MSAs) and homology-based data (HDB), are now at the core of modern structural bioinformatics. In practice, the full-resource AF2 implementation—leveraging complete homology databases and extensive computational power—has been established as the foundational tool for building the comprehensive AlphaFold Protein Structure Database (AFBD) hosted by the European Bioinformatics Institute (EBI) [8], which now provides structural coverage for the extensive UniProt protein repertoire supported by its performance in CASP benchmarks [9]. However, despite their impressive capabilities, it has been acknowledged that different versions or equivalent implementations can produce divergent results. This reveals an important knowledge gap: the extent to which such discrepancies influence the reliability of predictions for proteins whose structures remain experimentally unresolved [4]. This variation may have important biological implications, leading to different interpretations or conclusions depending on which tool is used particularly in contexts where structural models inform pathogenicity assessment, functional analysis of flexible or binding regions, or downstream applications such as molecular docking, variant prioritization, and structure-based drug design.

Furthermore, the accessibility and usability of these tools vary greatly. While some implementations require powerful hardware and significant bioinformatics expertise (e.g. official AF2/AF3 implementations [7]), others such as ColabFold (CF) [10] offer more accessible alternatives but may differ in output and performance.

A second challenge arises in how to evaluate and compare the performance of these prediction tools. Global evaluation metrics—such as root mean square deviation (RMSD), template modeling score (TM-score), and local distance difference test (LDDT) [3, 11]—offer valuable yet inherently limited perspectives on model accuracy. These metrics often rely on superimposition with known structures and offer only a summary view of local accuracy [10, 11, 12]. This limitation is further amplified by the presence of intrinsically disordered regions (IDRs), which are frequently missing from experimental structures but always modeled by prediction tools. As a result, standard superposition-based comparisons can be biased or misleading, particularly in unresolved proteins where IDRs constitute a substantial fraction of the sequence. As such, they can obscure critical differences at the residue or domain level, particularly in regions that matter most for downstream analyses, such as active sites or binding interfaces. For unresolved proteins this poses a major challenge [13]: how can one trust or select among predictions when standard metrics fail to capture local inaccuracies or biases? Together, these issues reveal a broader unaddressed challenge: despite the rapid expansion of AI-based predictors, there is currently no robust framework for systematically comparing their outputs when no experimental structure is available. To address these issues, we propose a dual-pronged solution. First, we perform a systematic comparison of different structure prediction tools against a reference one selected as the computational baseline. Initially the full-resource AF2 implementation, resembling the version used to generate the AFBD, was used as our primary point of comparison. This allows us to assess the concordance and reliability of more accessible tools, such as local implementations or web-based ones.

This framework was developed by using the SLC5A transmembrane protein and its 180 single amino acid variants (SAAVs) as a discovery dataset [14]. To ensure the robustness of our findings, we validated our approach across two proteins of the same family (SGLT1 and SGLT2) with 56 SAAVs [15] and over a diverse repertoire of seven additional proteins—varying in size, fold, and function—originally predicted by the EBI using the official Google DeepMind version of AF2 and 13 predicted variants [8, 16]. This strategy identifies precisely where and how these tools diverge, providing biologically relevant insights that empower users to select the most appropriate tool based on their specific research needs and available computational resources. Second, we introduce a novel per-residue evaluation framework, inspired by Bland–Altman analysis and multivariate projection techniques that allows for a deeper and more informative assessment of structural predictions. This approach moves beyond traditional global metrics to capture local discrepancies and highlight the specific protein regions where predictors differ. We demonstrate that this strategy not only reveals hidden weaknesses in current models but also enables the identification of functionally equivalent predictions, which is especially valuable when no ground truth is available [13, 17].

Together, this work provides a robust foundation for improving the interpretability, comparability, and reliability of protein structure prediction tools in real-world scenarios—particularly those involving novel or unresolved proteins [13, 17, 18].

## Materials and methods

### Prediction methods

Seven protein structure prediction tools were evaluated, including different implementations of AF2, OpenFold (OF), AF3, web server [7]), and CF. These predictors differ in algorithm setup, MSA tools, and homology database size. They were deployed and used as provided according to the author instructions with default parameters.

AF2 [2, 18] and OF [19] were executed using the AWS Batch Architecture for Protein Folding and Design (APFD) [20, 8] with two different Protein HDB configurations, the full (**AF2**_**F**_, **OF**_**F**_) and the reduced version (**AF2**_**R**_, **OF**_**R**_) and using the jackhmmer MSA search tool. In addition, the **AF2** Version 2.3.2 with reduced databases was installed and run on an A30 Nvidia GPU using Jackhmmer for MSA construction using the reduced database, dated July 2023, to minimize computer resource consumption, named AF2 local (AF2_L_), and the CF, Version 1.55 (April 2024), which uses MMseq2 and a custom MSA database hosted online and updated in real time was also used. Predictions were executed in Google Colab via the AlphaFoldXplore interface, which enables rapid batch submission of sequences and automatic download of results [21]. Finally, AlphaFold3 (May 2024) was accessed through the DeepMind web server.

To systematically evaluate the impact of different computational setups, we structured our benchmark across three primary axes of an ablation-inspired analysis (Table 1). This approach allows us to disentangle the effects of diverse models configurations and implementations within the available predictors: (i) HDB Scale: comparing full versus reduced databases while maintaining the MSA tool (Jackhmmer) (AF2_F_ vs. AF2_R_/AF2_L_); (ii) MSA Search Strategy: contrasting the traditional Jackhmmer search against the MMseq2 MS-Engine (CF); and (iii) Model Architecture and Paradigm: evaluating differences between AF2, the OF implementations (OF_F_/OF_R_), and the diffusion-based AF3.

**Table 1:** Overview of protein structure prediction methods and ablation-inspired analysis.

**Strategy**	**Predictor**	**Version and hardware platform**	**MSA tool and database**
HDB Scale	AF2_F_	v2.3.2/AWS (G4dn.xlarge)	Jackhmmer/full HDB
	AF2_R_	v2.3.2/AWS (G4dn.xlarge)	Jackhmmer/reduced HDB
	AF2_L_	v2.3.2/Local (NVIDIA A30)	Jackhmmer/reduced HDB
MSA tool	CF	v1.5.5/Google Colab (T4/L4)	MMseq2/ColabFoldDB
Model architecture	OF_F_	v1.0.0/AWS (G4dn.xlarge)	Jackhmmer/full HDB
	OF_R_	v1.0.0/AWS (G4dn.xlarge)	Jackhmmer/reduced HDB
	AF3	v3.0.0/Google/DeepMind Server	Internal/internal HDB

Comparison of protein structure prediction strategies, including versioning, hardware platforms, homology protein sequence database (HDB) (full or reduced) and multiple sequence alignment (MSA) configurations across various AlphaFold-based (AF) and OpenFold-based (OF) workflows. AF2_F_/AF2_R_/AlphaFold2 full/resource HDB version on AWS platform; AF2_L_ local AF2 implementation; CF, ColabFold (v1.5.5); AF3, AlphaFold 3; OF_F_**/**OF_R_, OpenFold full/reduced HDB.

### Protein data

To evaluate the robustness and consistency of structural predictions across varying predictive frameworks, we organized our analysis into three scenarios:

#### Discovery and development: Establishing the benchmark

The framework was developed using the Sodium/Iodide Symporter (NIS/SLC5A5), predicting by all models (see Table 1) and analyzing the wild-type (WT) sequence and 180 single nucleotides variants [14]. In this primary scenario, the full implementation of AF2 (v2.3.2) deployed on AWS infrastructure (AF2_F_) was established as the reference method mirroring the protocol used for the AFBD. It was considered as our “computational baseline” or competing method to evaluate the performance and divergence of all other predictors.

#### Intra-family validation: The competing method approach

To ensure the findings were consistent within a structural fold, we analyzed SGLT1 and SGLT2 and their respective variants [15] yielding 58 protein structures in total. In this stage, we transitioned our reference to the best-performing competitor against AF2_F_ identified in Scenario 1.

#### Extended generalization

Finally, to demonstrate the generalizability of our framework across the proteome, the dataset was expanded to a wide repertoire of seven proteins with different sizes, structures, and functions (Table 2). The included proteins provide a wide range of structural behavior, with BRAF showing the highest proportion of loops and IDRs, followed by ERBB2, TP53, CALR, GRK2, IDH1, and GNB1 as the most structured ones. Additionally, a total of 13 SAAVs were included, comprising BRAF (4), TP53 (4), and IDH1 (5). This set of proteins represents: (i) globular enzymes, (ii) transmembrane receptors, and (iii) a range of IDR content and protein compactness. For this set, the canonical predictions available from the AFBD [8] were used as the baseline and contrasted against their counterparts predicted with AF2_L_, CF, and AF3 [7].

**Table 2:** Overview of the evaluated protein dataset for framework validation of the different sets of proteins and variants.

**Target group**	**Protein(s)**	**Wild-type accession (Uniprot/AFDB)**	**Variants studied (SAAV)**	**Biological function**
Discovery set	NIS (SLC5A)	Q92911	WT + 180 SAAVs	Sodium:iodide symporter
Intra-family validation set	(SGLT1/2)	P13866, P31639	WT + 29 SAAVs (Both)	Sodium:glucose symporter
Extended validation set	BRAF	AF-P15056-F1-v6	WT + 4 SAAVs	Soluble kinase
	ERBB2 (HER2)	AF-P04626-F1-v6	Only WT	Receptor tyrosine kinase
	IDH1	AF-Q0QER2-F1-v6	WT + 5 SAAVs	Metabolic enzyme
	TP53	AF-P04637-F1-v6	WT + 4 SAAVs	Transcription factor
	GNB1	AF-P62873-F1-v6	Only WT	G-protein subunit
	GRK2	AF-P25098-F1-v6	Only WT	G-protein-coupled kinase
	CALR	AF-P27797-F1-v6	Only WT	Endoplasmic reticulum chaperone

Abbreviations: SAAV, single amino acid variants; WT, wild-type; AFDB, AlphaFold Protein Structure Database.

### Experimental design

#### Discovery and development phase

Since the AFDB primarily utilizes the official AF2 implementation, validated by its performance at CASP14 [9], we established here the full-resource implementation of AF2 (v2.3.2) deployed on AWS infrastructure (designed AF2_F_ in Table 1) as the computational baseline for the development step. To simulate a real-world scenario, where experimental resolved structures are frequently unavailable or scarce, we predict the structures for NIS (SLC5A) protein and all its variants using the suite of predictors detailed in Table 1. This phase allows developing our framework to systematically evaluate the inter-method agreement between the chosen reference model (AF2_F_) against less resource-intensive, different AI libraries, and different AI design predictor models (Table 1). In this sense, it is possible to determine which of them may be used instead of AF2_F_.

#### Confidence-based superimposition strategy

Due to the fact that inherently flexible (IDRs) frequently fail to produce interpretable electron density in X-ray crystallography or remain unresolved in cryo-EM density maps, experimental structures are often represented as a subset of the full-length protein sequence. Consequently, these segments appear as gaps in the final atomic model, despite being biologically present in the protein. In contrast, computational predictors generate 3D models of the entire sequence but with different per-residue confidence. To optimize protein superimposition and ensure a faithful comparison, global superposition, using all shared Cα atoms, and confidence-based superposition (CS), limited to residues with $\mathrm{pLDDT}\ge Trh$, with $Thr=\left\{50,70,\mathrm{and}\ 90\right\}$ in the predicted model were evaluated using NIS WT experimentally resolved protein.

#### Validation

After the selection of the AF2_F_ alternative predictor (discovery phase), the SGLT1/2 proteins (NIS family proteins) and their variants were used to evaluate the framework’s performance by predicting all the proteins by AF2_L_, CF, and AF3. Then, to further validate them in a broader scenario, the extended dataset was downloaded from the EBI web site hosting the AFDB [8] (see Table 2).

#### Prediction sensitivity to variants

Finally, the proposed benchmark was applied to evaluate the predictors sensitivity to single amino acid substitutions by comparing predicted WT structures against their corresponding variants counterparts (SAAVs) on subset of proteins, quantifying how different predictor alternatives are impacted by point mutations.

### Data acquisition and preparation

All the evaluated predictors provide atomic coordinates (*x*, *y*, *z*) stored in pdb files [6]. The pTM-scores, an estimate of the overall reliability of the predicted global fold, were recorded for each predicted protein (WT and its variants) across multiple predictors [7]. These data were processed using the Python-based library FoldComparative [22].

Confidence metrics (i.e. pLDDT per-residue local confidence estimate based on the LDDT-Cα metric [11]) were obtained directly from the pdb files predictors outputs. The predicted TM scores (pTM-score) were retrieved from the predictors outputs when available and used as global metrics [23, 8].

Since the AFBD does not provide pTM-scores for its deposited models, the DeepUMQA tool [23] was used to estimate them to ensure metric consistency across the entire dataset.

### The confidence matrices (*P*)

For each structure prediction method “*X*” and for each protein set (NIS, SGLT1, SGLT2), pLDDT scores were extracted to build a confidence matrix ${P}^X$. Each matrix had dimensions $N\ x\ L$, where *N* corresponds to the number of protein variants (the WT plus the mutated versions) and *L* holds for the protein length (see Table 2).

Each element ${p}_{v,r}^X$ of the matrix represents the pLDDT confidence score predicted by method *X* for residue “${r}^{"}\ (1..L)$ and protein variant ${}^{``}{v}^{"}\ (1..N)$. The general structure of the matrix is:


(1) \begin{equation*} {P}^X=\left[\ {p^X}_{v,1},{p^X}_{v,2},\dots .,{p^X}_{v,r},\dots .,{p^X}_{v,L}\ \right] \end{equation*}

To summarize the confidence across residues for each variant, we computed the row-wise average of ${P}^X$, resulting in a vector we denote as 


(2) \begin{equation*}\bar{P}_v^X = \frac{1}{L} \sum_{r=1}^{L} p^X_{v,r}\end{equation*}

This vector captures, for each variant, the overall structural confidence predicted by method *X*. Finally, the individual matrices were concatenated to form the comprehensive confidence matrix ${P}_k$ for $k=\mathrm{NIS},\mathrm{SGLT}1,\mathrm{SGLT}2$.

#### The confidence difference matrices (*M*)

To assess the differences in per-residue confidence scores between a reference method and each alternative predictor “$X,{}^{"}$ a confidence difference matrix ${M}^X$is built. The matrix retains the same dimensions as the original confidence matrices ${P}_{\left(V\times R\right)}^X$ (Equation 1), where “*v*” indexes the protein variants (1…*N*) and “*r*” the residues (1…*L*). Each element of ${M}^X$ is defined as: 


(3) \begin{equation*} {m}_{v,r}^X={p}_{v,r}^{Ref}-{p}_{v,r}^X \end{equation*}

Here, ${p}_{v,r}^{REF}$ is the pLDDT score for residue “*r”* of variant “*v”* predicted by the chosen reference method, and ${p}_{v,r}^X$ is the score predicted by method *X*. This formulation ensures that ${M}^X$ directly reflects the residue-level confidence deviations of each method *X* with respect to the chosen reference within the same protein group. All matrices ${M}^X$ were vertically concatenated to form a global matrix: 


(4) \begin{equation*} {M}^{X}=\left[\ {m^{AF2-X}}_{v,1},{m^{AF2-X}}_{v,2},\dots .,{m^{AF2-X}}_{v,r},\dots .,{m^{AF2-X}}_{v,L}\ \right] \end{equation*}

To evaluate this metric globally, we calculated the mean difference across residues for each protein variant: 


(5) \begin{equation*} \overline{M_x}=\frac{1}{L_K}{\sum_{r=1}^L}{m}_{v,r}^X\kern0em \end{equation*}

where ${L}_k$ denotes the protein length for the group *k =* NIS, SGLT1, SGLT2. The resulting distribution of $\underset{\_}{M_x}$values were used for comparing the overall deviation across methods.

#### Structural difference matrix (*D*)

For each predictor *X*, we constructed a structural difference matrix ${D}^X$, where each element represents the Euclidean distance 


(6) \begin{equation*}d(C\alpha_{v,r}^{AF2}, C\alpha_{v,r}^X) = \sqrt{(x_{v,r}^{AF2} - x_{v,r}^X)^2 + (y_{v,r}^{AF2} - y_{v,r}^X)^2 + (z_{v,r}^{AF2} - z_{v,r}^X)^2}\end{equation*}

between the aligned $\mathit{\mathrm{C}\alpha }$ atoms (extracted from the pdb files [6]) of the predicted structures from AF2_F/L_ and predictor *X*, i.e.:


(7) \begin{equation*} {D}^X=\left\{d\Big({\mathrm{C} \alpha}_{v,r}^{AF2},{\mathrm{C} \alpha}_{v,r}^X\right\},\forall X\ne \mathrm{AF}2 \end{equation*}

All structures were aligned using the Superimposer module from Biopython [24], **CS strategy**. Following the results of our optimization procedure (see Section 3.1), only residues with a baseline *pLDDT≥ 70* were utilized to compute the initial alignment, ensuring that structurally stable regions drive the superposition. Each resulting matrix *D* had dimensions 181 × 643 and was vertically concatenated into: 


(8) \begin{align*} {D}_k=\left[{D}^{A\mathrm{F}2-\mathrm{CF}},{D}^{\mathrm{AF}2-\mathrm{AF}{2}_{\mathrm{L}}},{D}^{\mathrm{AF}2-\mathrm{AF}{2}_{\mathrm{R}}},{D}^{\mathrm{AF}2-\mathrm{AF}3},{D}^{\mathrm{AF}2-\mathrm{O}{\mathrm{F}}_{\mathrm{F}}},{D}^{\mathrm{AF}2-\mathrm{O}{\mathrm{F}}_{\mathrm{F}}}\right]^{T} \end{align*}

All matrices and structural comparisons were computed using dedicated functions implemented in the FoldComparative Python library [22].

### Benchmark metrics and comparison tests

To globally assess the agreement between predicted structures, we selected three benchmarking metrics that are widely recognized within the scientific community, including those commonly employed in structure prediction assessments such as CASP [9].

#### $\mathrm{Mean}\Delta \mathrm{pLDDT}$

Calculated from the confidence difference matrix ${M}^{X}$ (Equation 4), this metric evaluates how much a predictor’s confidence scores deviate from a reference implementation (AF2_F_/AF2_L_). This differential approach provides a more concrete understanding of the extent of improvement (or divergence) offered by alternative implementations.

#### Root mean square deviation

Calculated from the distance matrix ${D}^X$ (Equation 7), this metric represents the average deviation between equivalent atoms in aligned residues. Lower values (measured in ångströms, Å) indicate better structural superposition.

#### TM-score

Also derived from the ${D}^X$ matrix, this metric evaluates global structural similarity based on the alignment of $\mathrm{C}\mathrm{\alpha}$ atoms. It ranges from 0 to 1, where values closer to 1.0 reflect a higher degree of structural agreement between the compared models [7].

### Per-residue profile analysis

#### Per-residue agreement analysis

To capture local differences that global metrics may obscure, a residue-level analysis was performed.

- **Confidence Bias Analysis (M–P plot):** We utilized the **Bland–Altman analysis** to assess systematic biases in prediction confidence [17]. This plot represents the difference in per-residue pLDDT confidence differences—derived from the **Matrix**  $\boldsymbol{M}$ (${\mathrm{pLDDT}}_{\mathrm{AF}2}-{\mathrm{pLDDT}}_X$)—against the mean of both measurements, allowing for the direct identification of overestimation or underestimation across different confidence intervals. To highlight non-linear trends, a **LOESS regression curve** was incorporated into each plot.
- **Distance–pLDDT (D–P) plot for Structural Deviation Analysis:** To evaluate structural agreement, we propose a plot inspired by the Bland–Altman approach. This represents the 3D Euclidean distance between the $\mathrm{C}\mathrm{\alpha}$ coordinates per residue (*Y*-axis) against the pLDDT of the reference predictor (*X*-axis). This plot reveals whether structural differences are associated with low- or high-confidence regions, adding structural context to the pLDDT metric.

This dual methodological proposal refines traditional global metrics by incorporating a residue-level analysis. Furthermore, this strategy is systematically applicable to comparative studies with multiple implementations, enabling the detection of differences in local predictive capacity with high resolution.

#### UMAP projection

The UMAP technique [25] was applied to project per-residue confidence profiles (high-dimensional vectors) into a two-dimensional visual space. This allows for the identification of patterns and clusters, revealing similarities, or characteristic “signatures” in the confidence profiles generated by each predictor.

Together, these analyses refine global metrics by exposing functionally relevant divergences in specific regions.

#### Statistical analysis

All statistical analyses were performed using non-parametric tests. Group-level comparisons were assessed using the Kruskal–Wallis test, followed by pairwise evaluations using the Wilcoxon signed-rank test. To account for multiple testing across families of pairwise comparisons, *P*-values were adjusted using the Benjamini–Hochberg procedure to control the false discovery rate (FDR). Adjusted *P*-values (*q*-values) are reported throughout the manuscript, and statistical significance was defined as *q* < 0.05. In addition to significance testing, effect sizes were calculated for all primary comparisons to provide a quantitative measure of the magnitude of observed differences. Full statistical outputs, including raw *P*-values, *q*-values, and effect sizes, are provided in Supplementary Tables S1–S4.

## Results

### Significant differences between predictors are revealed by global and per-residue predicted metrics

**Figure 1:**
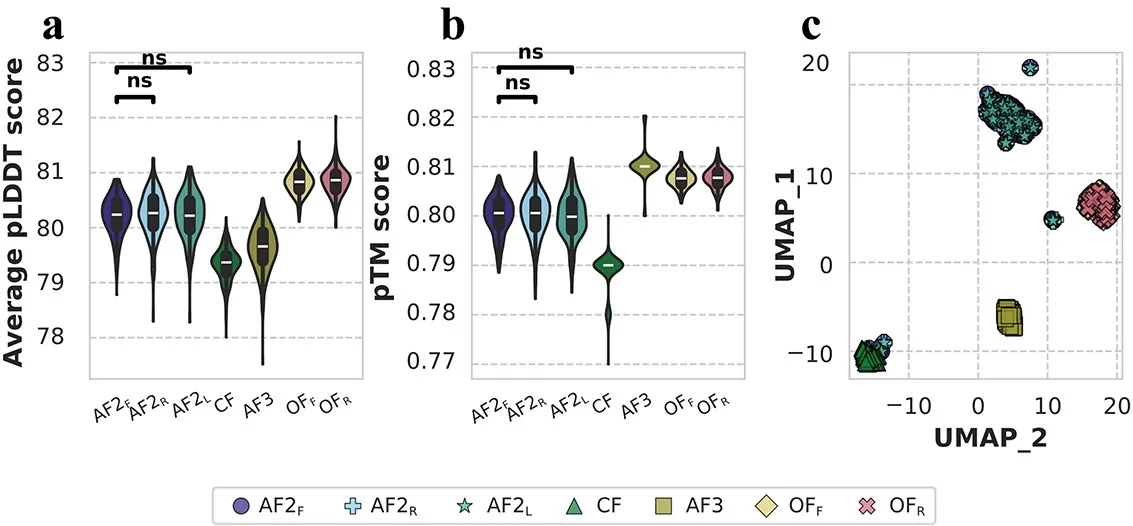
Comparison of prediction confidence between different structure predictors. (a) Distribution of average pLDDT scores per protein for each predictor, AlphaFold2 implementations (AF2_F_, AF2_R_, AF2_L_), ColabFold (CF), AlphaFold3 (AF3), and OpenFold implementations (OF_F_, OF_R_). (b) Distribution of pTM-scores for the same set of predictions. Only non-significant (“ns”) differences are explicitly labeled. (c) UMAP projection of the per-residue pLDDT profiles, showing distinct clustering by prediction tool irrespective of protein variant.

Figure 1a summarizes the distribution of average pLDDT values ($\overline{P_v^X}$, Equation 2) for each predictor$X\in \{ AF{2}_F, AF{2}_R, AF{2}_L, CF, AF3,O{F}_F, O{F}_R\}$ across all protein variants *(v)*. It can be observed that for NIS protein CF consistently yielded significantly lower average pLDDT values compared to the other methods (Wilcoxon rank-sum test *q<0.001*, FDR-corrected), suggesting the impact of the MSA algorithm when compared with AF2 implementations using the jackhmmer MSA search engine. This divergence is further supported by a large effect size (*d* = 2.63, Table S1). On the other hand, OF produced the highest pLDDT scores (*d* = 2.10) while AF3 ranked between CF and the AF2 implementations, suggesting performance differences regarding the predictor’s algorithms. Notably, no significant differences were observed between the AF2_F_ baseline and its reduced or local implementations (AF2_R_, AF2_L_; *q >* 0.05).

When considering pTM (Fig. 1b), a similar trend was observed, although AF3 achieved the highest values for this metric (*q* > 0.001; *d* = 2.84)*,* altering the interpretation compared to pLDDT. This discrepancy between global metrics leads to an inconsistent interpretation, making it challenging to determine which method performs best based solely on summary indicators.

On the other hand, multidimensional projection (UMAP) [25] (Fig. 1c) of the per-residue confidence matrices ${P}^X$(Equation 1) clearly depicts that pLDDT profiles tend to be similar between protein variants irrespective of the used Homology database (see Table 1 HDB Scale) of AF2 and OF, yet remain highly dissimilar regarding the MSA search engine (i.e. CF, AF2, AF3, and OF).

Altogether, these findings indicate that confidence profiles differ significantly across implementation setups. While global metrics provide a simplified and often contradictory view, UMAP results show that each method generates distinct residue-level confidence patterns, even when predicting the same protein sequences. This highlights how architectural and algorithmic choices influence confidence estimates beyond what global metrics can capture, a finding supported by the large effect sizes observed in our standardized statistical framework (Table S1).

### Lightweight AF2 with jackhmmer search engine performs similarly to AF2 with high computational and HDB resources

Due to its performance on CASP 14 [9] and its use in developing the AFBD, we deploy AF2 with full computer hardware capabilities (G4 AWS instances) and maximum HDB support (named AF2_F_) on the AWS Batch APFD platform. This AF2_F_ was used as the computational baseline predictor or reference to compare all the other models/implementations against it by using NIS and its variant sequence proteins (the discovery and development step).

To quantify these relative differences at the residue level, we constructed a confidence difference matrix ${M}^{X}$ (Equation 4) for each evaluated configuration (i.e. *X* = AF2_L_, AF2_R_, CF, AF3, OF_F_, OF_R_) in comparison to AF2_F_ baseline. Each row of these matrices contains the vector of per-residue pLDDT differences for a specific variant NIS protein.

**Figure 2:**
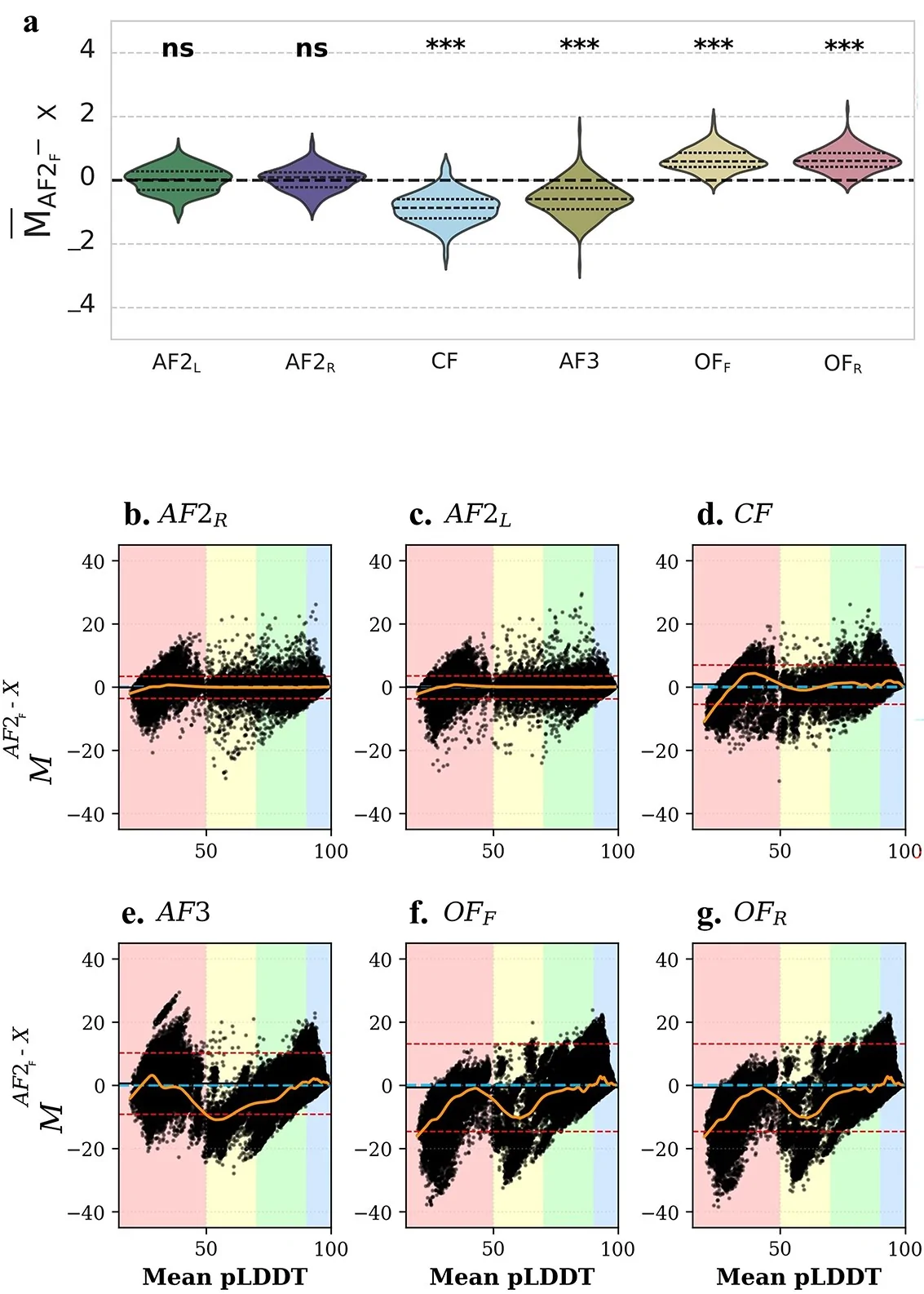
Global- and residue-level comparison of predictors relative to the full-resource AF2_F_ (as the computational baseline reference). (a) Mean per-protein pLDDT differences *M* (${\mathrm{pLDDT}}_{\mathrm{AF}{2}_{\mathrm{F}}}-{\mathrm{pLDDT}}_X$) for each comparison by method (*X*: AF2_R_, AF2_L_, CF, AF3, OF_F_, OF_R_). Boxplots show median and interquartile ranges; asterisks (*) indicate distributions significantly different from zero (paired Wilcoxon test, *P* < .05). (b–g) Bland–Altman (M–P) plots comparing per-residue confidence. The *x*-axis represents the mean pLDDT scores between the reference and method *X*, while the *y*-axis shows the per-residue difference *M*(${\mathbf{pLDDT}}_{\mathbf{AF}{\mathbf{2}}_{\mathbf{F}}}-{\mathbf{pLDDT}}_{\boldsymbol{X}}$). Solid lines denote mean differences and red curves show LOESS trend fits. Background colors indicate AF2 confidence intervals: Very low (<50), low (50–70), confident (70–90), and very high (≥90).

Figure 2a represents the distributions of the row averages of each $\overline{M_x}$ (Equation 5), providing a simplified, global overview. Based on these distributions alone, CF and AF3 tend to provide, on average, significantly lower pLDDT scores than AF2_F_ meanwhile OF yields consistently higher values. These differences are statistically significant (Wilcoxon paired test, compared to zero as the baseline). In contrast, the alternative AF2 configurations, addressing the HDB Scale comparison (AF2_L_ and AF2_R_), do not show significant differences in average pLDDT differences. However, relying solely on mean differences limits the analysis, as it does not reveal where in the protein these discrepancies occur or whether they follow consistent patterns.

To address this limitation, we used a per-residue comparison based on Bland–Altman plots (M–P plots), where the value of the ${M}_k^{AF{2}_F-X}$ is plotted against the sorted pLDDT of the chosen reference model, in this case the AF2_F_ as a computational baseline. This approach allows us to examine whether per-residue discrepancies are randomly distributed or show systematic trends. We found that both reduced AF2 versions (AF2_L_ and AF2_R_) yield pLDDT differences randomly centered around zero, indicating good agreement with the full-resource AF2 predictions (AF2_F_) thus suggesting that lower computational and database resources can be used instead of the full architecture thus diminishing costs. Conversely, CF, which differs from the MSA Search Strategy (MMseq2 vs. Jackhmmer) shows a higher dispersion and a consistent greater pLDDT values at ranges pLDDT ⪅ 35 and 50 ≤ pLDDT ≤ 70. To further explore whether these differences depend on prediction confidence, we categorized residues according to the quality intervals defined for AF2 [9]: very low confidence (pLDDT < 50), low confidence (50 ≤ pLDDT < 70), confidence (70 ≤ pLDDT < 90) and high confidence (plDDT ≥ 90) a Bland–Altman like plot is recovered [17].

Figure 2b–g shows that, with the exception of AF2_L_ and AF2_R_, all predictors display clear biases across these intervals. In this context, overestimation refers to predicting a higher pLDDT value than the AF2_F_, while underestimation means predicting a lower pLDDT value than the AF2_F_. Loess trend lines indicate that CF tends to overestimate pLDDT when the baseline score (AF2_F_) is below 40, underestimate it between 40 and 50, and then overestimate again in low confidence regions. Surprisingly, AF3 generally underestimates pLDDT when AF2_F_ < 40 and overestimates it in the range 40 ≤ pLDDT_AF2_ ≤ 90 suggesting differences regarding the differences in the AlphaFold designs between versions 2 and 3. Finally, both OF implementations, in this case contrasting model implementation differences regarding programing libraries (tensorflow vs. pytorh), show a pattern similar to CF, overestimating AF2_F_ scores in very low and low-confidence regions and underestimating them in pLDDT_AF2_ > 75 regions.

These results highlight that systematic biases and local patterns remain hidden in global summaries. Importantly, the strong agreement between AF2_L_, AF2_R_, and AF2_F_ supports the use of lightweight AF2 implementations, such as the one presented here running on a mid-range NVIDIA 30-series GPU (AF2_L_), for efficient and reliable protein structure prediction without loss of technical consistency. The M–P plots also suggest that the most pronounced discrepancies are attributed to differences in the MSA search algorithm and (CF) and predictor modeling paradigm (AF3 and OF) (see Table 1).

### High confidence Cα-based superimposition improves protein structural similarity metrics

The above results suggest that AF2_L_ may serve as an effective low-resource tool for protein structure prediction. However, decisions based solely on predicted quality metrics may not be conclusive, raising the question of whether the per-residue 3D spatial allocation shows comparable agreement in coordinate positioning. In this sense, accurate protein structure 3D coordinate superposition becomes crucial, and it depends on which residues are used for alignment. While residue selection for coordinate superimposition is straightforward in experimentally resolved proteins (it is done between its residues and the corresponding residues of the predicted protein), it becomes more complex with predicted structures due to the presence of the full sequence that can contain disordered regions (often missing in resolved protein structures). Thus, appropriate residue selection becomes crucial since it has been reported that the inclusion of predicted large IDR regions (more than 20 residues) negatively impacts the commonly used similarity metrics like RMSD or TM-score [13]. Table 3 shows structural similarity metrics (RMSD and TM-score) for NIS, comparing predicted models from all evaluated methods to the experimental structure using predicted residues with pLDDT values in the different confidence regions. Two alignment strategies were used: (i) global superposition, using all shared Cα atoms, and (ii) CS, limited to residues with pLDDT ≥ Trh with Thr = {50, 70, and 90} in the predicted model. Observations indicate that using residues satisfying pLDDT ≥ 70 yields higher similarity metrics (lowest RMSD and highest TM-scores) for all models and particularly significant better scores than global alignment across all methods. Although global RMSD differences between methods appear modest (Table 3), per-residue statistical analysis revealed consistent and statistically significant differences across all thresholds. However, effect sizes were generally small to moderate, particularly among top-performing models such as AF2_L_ and AF2_F_ (Table S2).Thus, this approach was adopted for all further comparisons to reduce the influence of uncertain regions.

**Table 3:** Structural similarity between predicted and experimental NIS models.

**Metrics**	**RMSD (Å)**	**TM-score**	**AAs**	**RMSD (Å)**	**TM-score**	**AAs**	**RMSD (Å)**	**TM-score**	**AAs**	**RMSD (Å)**	**TM-score**
pLDDT	0–100		≥50			≥70			≥90		
CF	2.572	0.715	500	2.561	0.718	489	**2.550**	**0.721**	332	2.564	0.720
AF2_F_	2.531	0.730	500	2.526	0.725	492	**2.511**	**0.730**	366	2.526	0.726
AF2_L_	2.511	0.725	500	2.504	0.728	493	**2.484**	**0.734**	367	2.504	0.730
AF2_R_	2.554	0.719	500	2.549	0.721	492	**2.532**	**0.726**	365	2.552	0.721
AF3	2.573	0.715	501	2.561	0.717	497	**2.563**	**0.717**	319	2.525	0.728
OF_F_	2.512	0.723	500	2.509	0.725	491	**2.503**	**0.728**	312	2.608	0.703
OF_R_	2.511	0.722	500	2.509	0.7254	491	**2.48**	**0.7267**	312	2.6075	0.7034

TM-score and RMSD were computed using: (i) global alignment (all shared Cα atoms), and (ii) confidence superposition (CS; pLDDT ≥ 70). The “AAs (CS)” column indicates the number of aligned residues under CS. CS consistently improved structural similarity. Compared methods: AF2_L_, AF2_F_, AF2_R_ (AlphaFold2); CF (ColabFold); AF3, OF_F_, and OF_R_ (OpenFold). Pairwise statistical comparisons of per-residue RMSD distributions between methods, including Wilcoxon signed-rank tests with Benjamini–Hochberg correction, are provided in Table S2.

The adoption of CS-based filtering not only improves absolute similarity metrics but also provides a more reliable framework for detecting subtle yet consistent differences between prediction methods.

### Predicted residue 3D positions significantly differ between predictors in very-low confidence regions associated with IDR loops

Having defined both the competing AF2_F_ predictor (i.e. AF2_L_) and its optimal superimposition approach, we proceed to validate our benchmark framework by using the chosen predictor (AF2_L_) as our new computational baseline resource against low cost predictor alternatives CF and AF3. Acknowledging that global metrics may mask where these differences are located in the protein sequence and structure, we propose the use of the Distance–pLDDT (D–P) plot to contrast the per-residue Euclidean distances $d\left({\mathit{\mathrm{C}\alpha}}_{v,r}^{AF2},{\mathit{\mathrm{C}\alpha}}_{v,r}^X\right)$ (Equation 6) against the corresponding pLDDT scores assigned by the AF2_L_ baseline.

**Figure 3:**
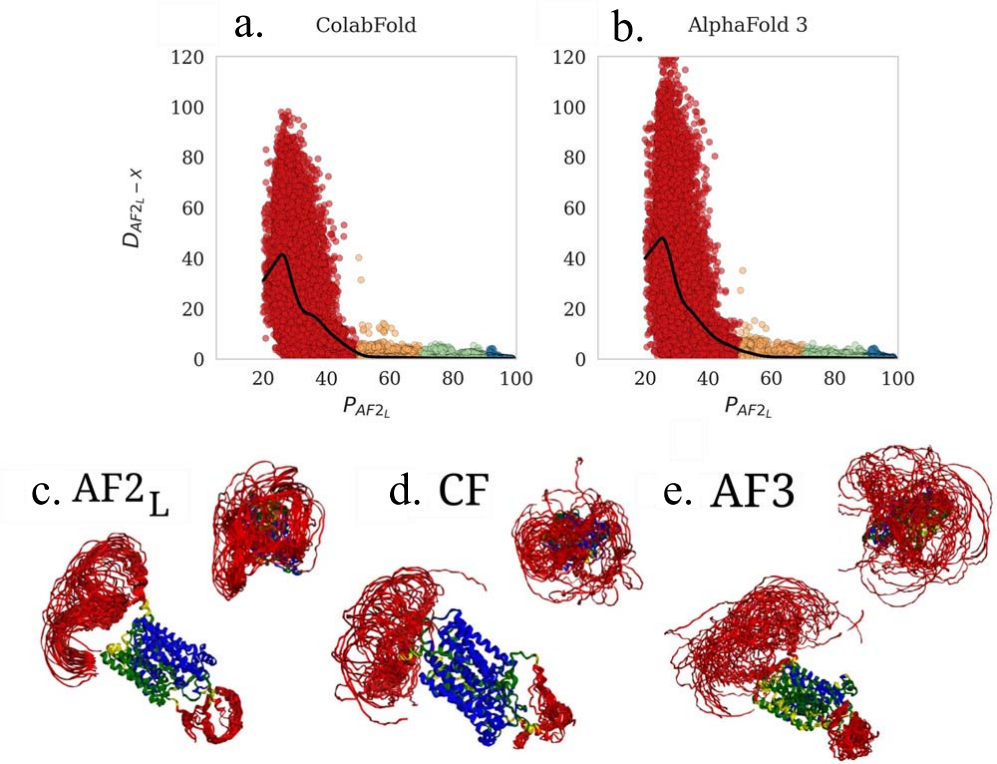
Structural divergence between predictors is mainly restricted to low-confidence disordered regions. (a and b) Per-residue cα distances between the AF2_L_, as the computational baseline, and ColabFold (CF, a) or AlphaFold3 (AF3, b), plotted against AF2_L_ pLDDT scores. Loess curves highlight deviation trends. (c–e) Representative 3D superpositions of the NIS protein using AF2_L_ (c), CF (d), and AF3 (e). Structural discrepancies are concentrated in very low-confidence regions (pLDDT < 50) with distances greater than 60 Å, while the high-reliability core shows high agreement among the different predictors.

Figure 3a and b shows the per-residual Euclidean distances for each residual “*r*” and for all predicted proteins against the different pLDDT_AF2_L__ confidence regions, where it is clearly depicted that the greatest difference emerges on the very low confidence regions. In addition, loess local regression curves suggest that, on average, AF3 tends to provide higher differences than CF. In Fig. 3c–e side and front views of the superimposed protein structures predicted by AF2_L_, CF, and AF3 are shown, with residues colored according to pLDDT-defined intervals. These regions correspond to intrinsically disordered protein (IDP) loops. Notably, the “clouds” formed by the predicted main loops appear broader in CF and AF3 compared to AF2_L_. These results shows how our comparison framework allows to identify the effects of MSA Search Strategy in CF and the impact of the different modeling paradigms (i.e. the diffusion-based model AF3), mainly in the IDRs compared to the Jackhammer-based AF2_L_ implementation.

### Extended validation of the benchmarking framework confirms consistent method-dependent patterns across diverse protein architectures

**Figure 4:**
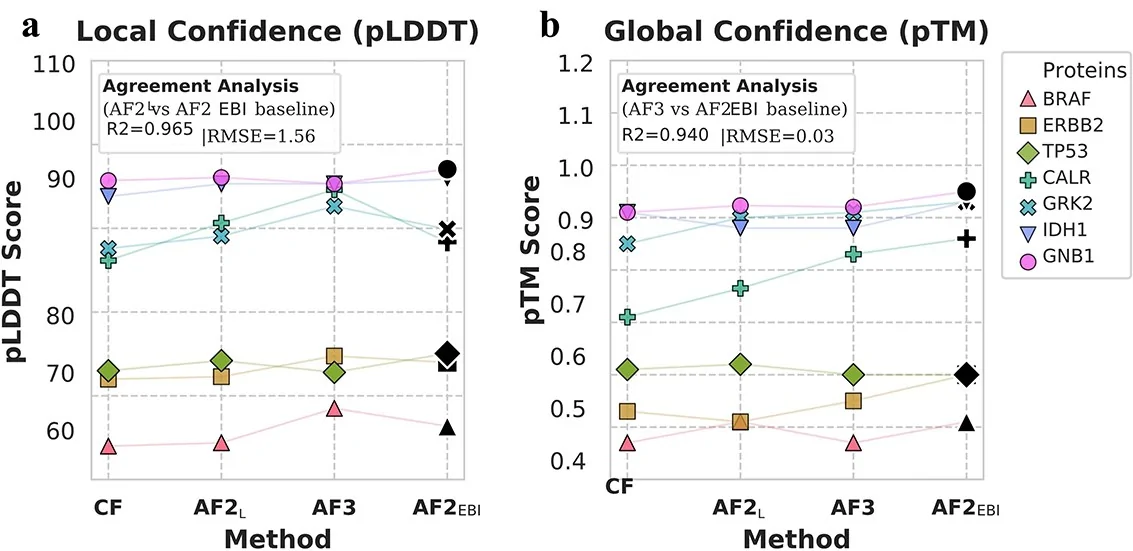
Evaluation of global and local confidence metrics across structurally diverse proteins. (a) Distribution of average local confidence (pLDDT) scores. Inset table displays agreement metrics (coefficient of determination, *R*^2^; and root mean square error, RMSE) for AF2_L_ against the reference. (b) predicted global fold reliability expressed as pTM-score for each method: CF; AF2_L_; AF3, and baseline structure from AFBD (AF2_EBI_) inset table displays agreement metrics for AF3 against the reference. For the variant-containing proteins (BRAF, TP53, and IDH1), individual points represent single-residue substitutions.

To further evaluate the generalizability of our benchmarking approach, it was applied to a set of proteins with distinct functional and conformational profiles (Table 2) providing a wide range of structural behaviors, varying in disordered region content and overall compactness. The PDB files of all the canonical proteins were retrieved from AFBD, hosted by the EMBL-EBI website [8]. These models, generated via AlphaFold Monomer v2.0 pipeline by Google DeepMind [2], were used in this case as the structural reference for the comparative framework. Figure 4a shows the average pLDDT values and Fig. 4b shows the pTM-scores of the predicted proteins. It can be observed that no consensus along the predictors can be achieved nor the potential source of the observed differences when comparing predictors consistent with our previous observations regarding global metrics discrepancies (Fig. 1a and b).

**Figure 5:**
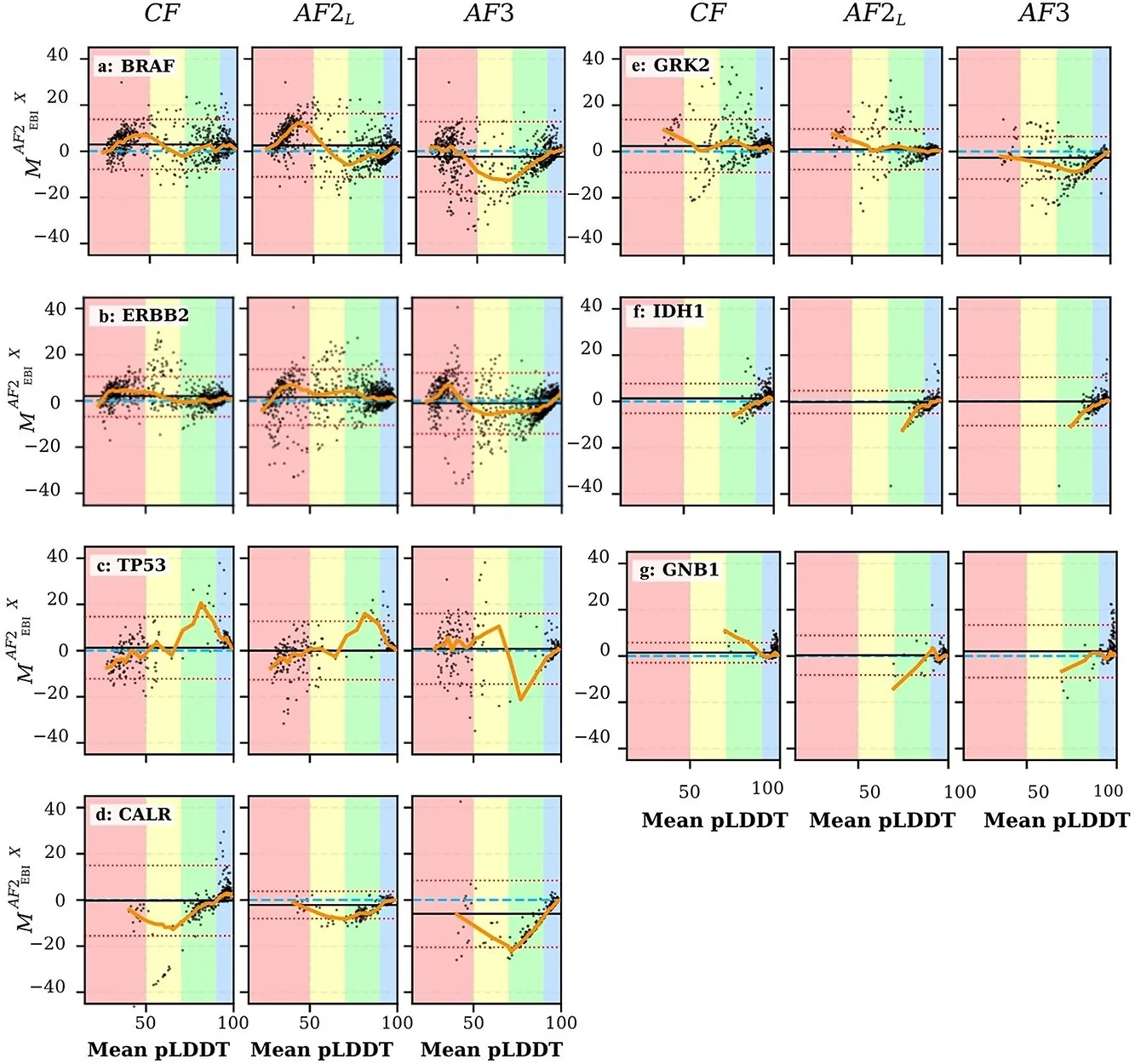
Bland–Altman (M–P) plots across the extended protein set showing per-residue agreement in pLDDT between the AF2_EBI_ baseline and alternative predictive methods. Differences are computed as (${\mathbf{pLDDT}}_{\mathbf{AF}{\mathbf{2}}_{\mathbf{EBI}}}-{\mathbf{pLDDT}}_{\boldsymbol{X}}$) against the mean of both scores, enabling visualization of systematic bias and dispersion patterns across confidence regimes. Background shading denotes AlphaFold confidence tiers (very low <50, low 50–70, confident 70–90, very high ≥90). Rows correspond to representative proteins: (a) **BRAF**, (b) **ERBB2**, (c) **TP53**, (d) **CALR**, (e) **GRK2**, (f) **IDH1**, and (g) **GNB1**, while columns show comparisons against CF, AF2L, and AF3. The curve indicates the LOESS-smoothed trend, highlighting local deviations from agreement.

**Figure 6:**
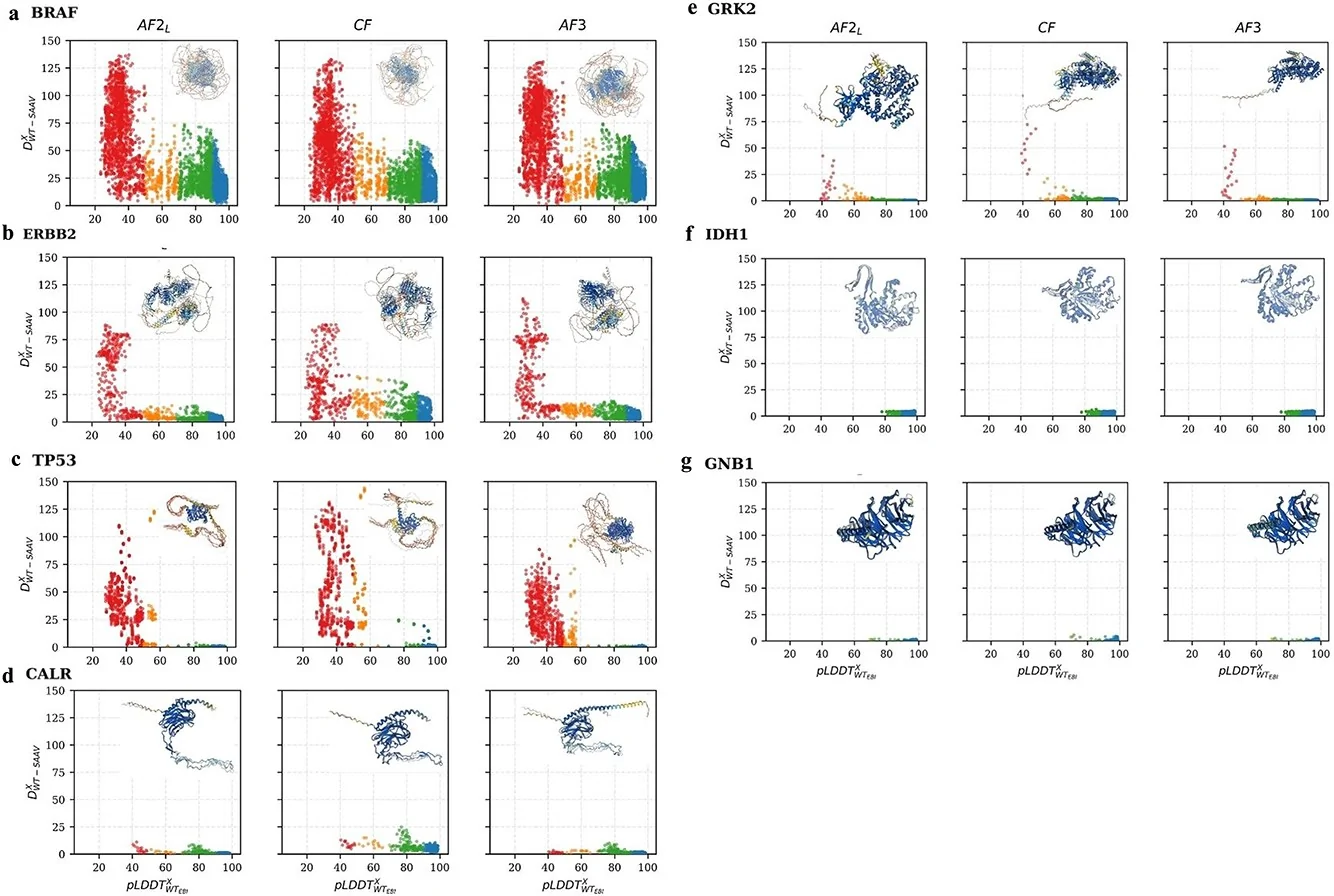
Distance–pLDDT (D–P) plots across the extended protein set illustrating the relationship between structural deviation and model confidence. Per-residue Euclidean distances (${D}^{\mathrm{AF}{2}_{\mathrm{EBI}}-X}$, in Å), computed after confidence-based structural superposition, are evaluated as a function of **AF2**_**EBI**_ pLDDT scores, enabling assessment of how prediction errors distribute across confidence regimes. Background shading denotes AlphaFold confidence tiers (very low <50, low 50–70, confident 70–90, very high ≥90). Rows correspond to representative proteins: (a) **BRAF**, (b) **ERBB2**, (c) **TP53**, (d) **CALR**, (e) **GRK2**, (f) **IDH1**, and (g) **GNB1**, while columns show comparisons against CF, AF2_L_, and AF3. Insets display corresponding 3D structural superpositions. The LOESS-smoothed curve highlights non-linear trends in the confidence–error relationship.

However, when using the proposed benchmark framework it is possible to observe that M–P plots (Fig. 5) and D–P plots (Fig. 6) clearly depict where the differences appear. In the case of M–P plots. The previous pattern emerges, showing that for proteins with IDRs, the main differences remain in the loops holding middle pLDDT confidence values. As expected, AF2-based models (CF and AF2_L_) exhibited higher structural similarity to the reference AFDB models. In contrast, the AF3 models showed the greatest deviations from the mean difference (indicated by dashed lines) when compared against the baseline structures from the EMBL-EBI repository [8].

Figure 5 illustrates that, upon superimposition, the most significant positional discrepancies between predictors are primarily localized in loop regions or terminal tails—areas typically associated with lower confidence scores. For well-structured and compact proteins (i.e. GRK2, IDH1, GNB1) all the proteins predicted by competing methods (CF, AF2_L_, and AF3) shows good agreement with the baseline AFDB structures retrieved from the EBI portal [8].

These results indicate that for a comprehensive analysis and understanding of protein structure predictors, the proposed benchmark framework provides valuable insights into the difference and agreement between competing protein structure predictors.

### Single amino acid substitutions significantly alter predicted loop residue positions in 3D space

**Figure 7:**
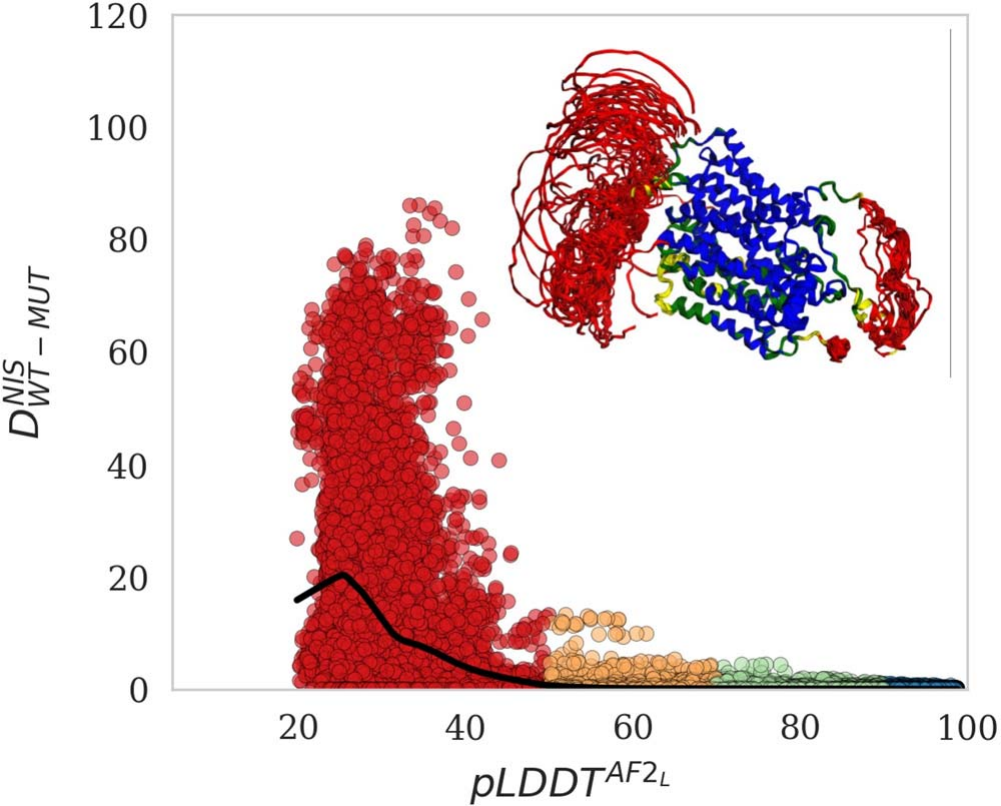
Structural impact of SAAVs on NIS. Distance–pLDDT (D–P) analysis of per-residue Cα deviations between WT and single amino acid variant (SAAV) models predicted with AF2_L_. Distances are evaluated relative to pLDDT confidence scores, allowing assessment of how local structural perturbations relate to model confidence. Background coloring follows AlphaFold confidence tiers (very low <50, low 50–70, confident 70–90, very high ≥90). Insets show the 3D NIS structure shaded by pLDDT, highlighting the spatial correspondence between high-deviation residues and disordered loop regions.

Recent advances extending AlphaFold-based frameworks, such as AlphaMissense [16], highlight a paradigm shift from static protein structure prediction toward the interpretation of sequence variants and their functional consequences. Given that only a small fraction of variants have been experimentally characterized, these approaches leverage structural context to infer pathogenicity. Motivated by these developments, we investigated how different prediction models capture mutation-induced structural variability, using NIS as a representative system. To achieve this, we employed a comparative approach, contrasting the mutated protein structures (SAAVs) against their respective WT predictions generated by each model. Notably, Fig. 7 illustrates the Euclidean distances between the predicted WT and mutated versions (SAAVs) of the NIS protein using AF2_L_ (additional results for others methods are available in Supplementary Fig. S1), with superimposed structural side views provided for visual context.

This approach highlights two primary observations: first, the largest Cα deviations are localized within protein loops, which correspond to regions of low-confidence coordinate predictions (pLDDT < 50). Second, AF3 appears significantly more sensitive to SAAVs compared to other predictors (Supplementary Fig. S1).

**Figure 8:**
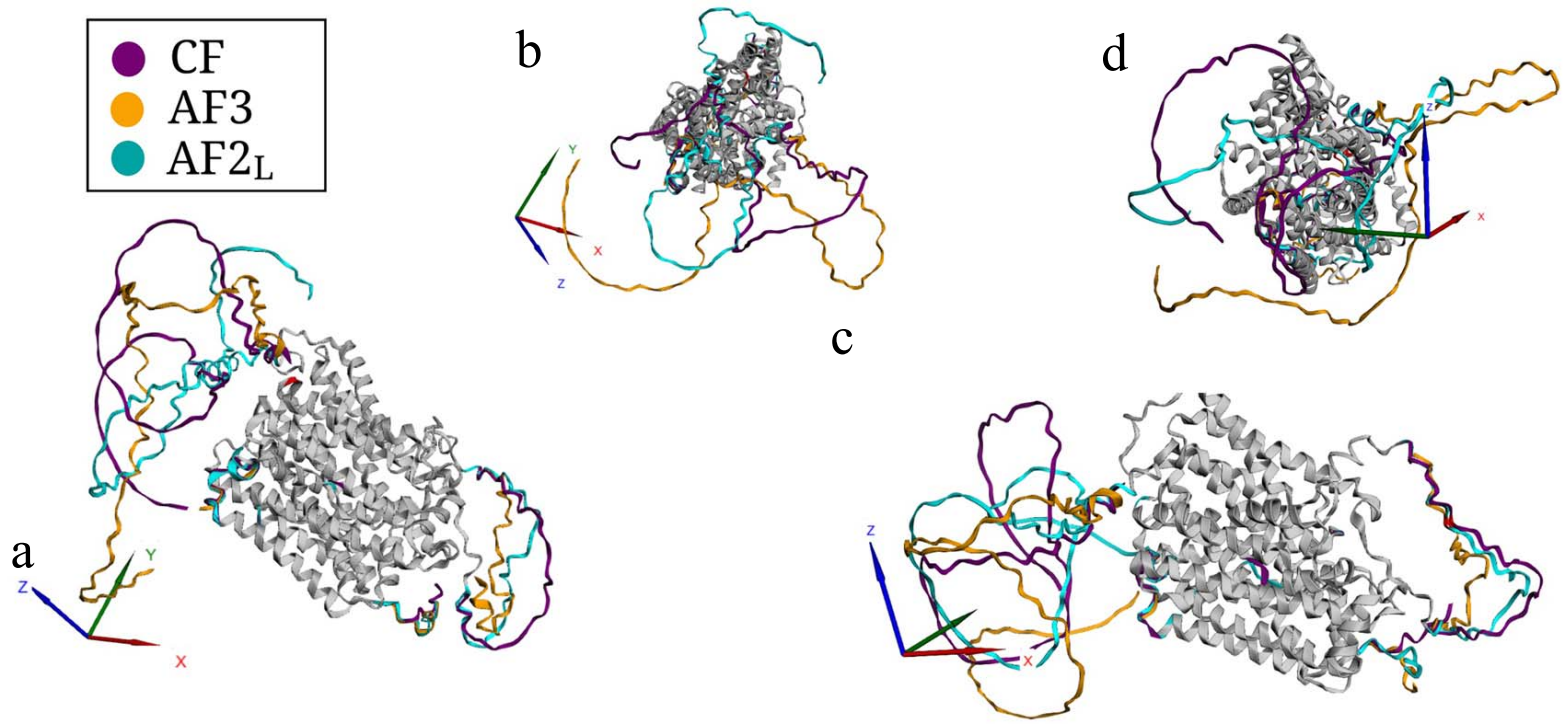
Structural impact of SAAVs on NIS. Side and frontal views of confidence-filtered structural superpositions (pLDDT ≥ 70) of NIS models predicted by CF (purple), AF2_L_ (cyan), and AF3 (amber) for representative single amino acid variants. (**a** and **b**) Arg124Asn, located in a high-confidence region, with the mutation site highlighted in red. (**c** and **d**) Arg506Thr, positioned within a low-confidence loop. Structural divergence is greater in the disordered region (IDRs) than in the structured core.

These findings suggest that the main effect of a SAAV on the predictors is linked to increase per-residue coordinate differences, particularly at residues located within IDRs (pLDDT < 50), and this effect arises regardless of the mutation site. Although the overall folds predicted by the models remain broadly consistent, the presence of a single mutation is sufficient to generate a detectable structural impact. This impact is observed not only when the variant lies within low-confidence loop regions but also when it occurs in highly conserved regions with high pLDDT values (>90), where the resulting perturbations propagate through the structure and are mainly reflected in the flexible loops. This pattern is clearly illustrated in Fig. 8, which compares the effects of variants located in different structural contexts. Specifically, Fig. 8a and b shows predictions for the Arg124Asn variant, located in a structurally conserved high-confidence region (pLDDT = 94.1), while Fig. 8c and d displays the Arg506Thr variant, located in a low-confidence loop region (pLDDT = 26.62).

**Figure 9:**
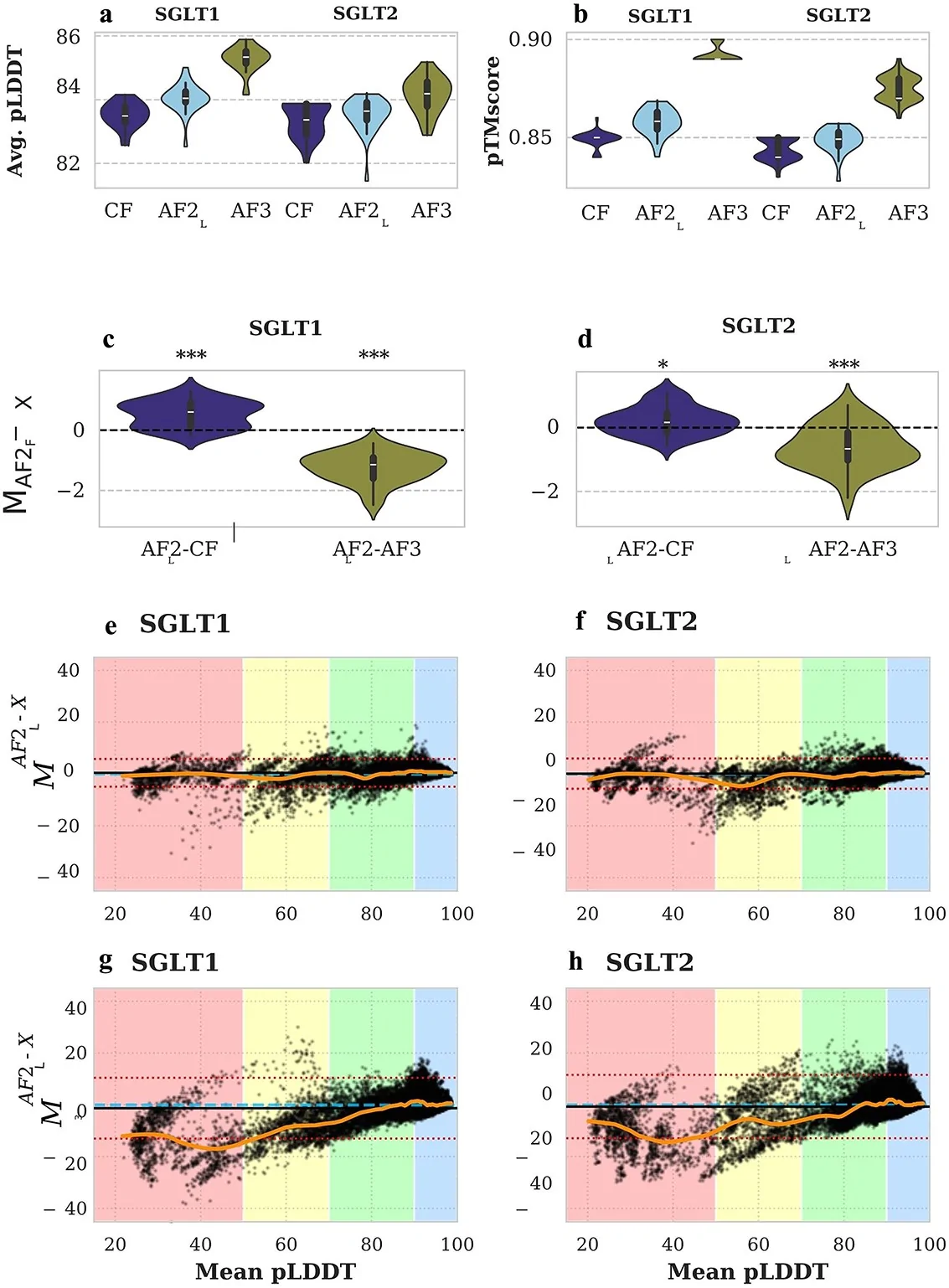
Comparative analysis of SGLT1 and SGLT2 structure predictions generated with ColabFold (CF), local AlphaFold2 (AF2_L_), and AlphaFold3 (AF3). Global confidence metrics (**a** and **b**) show average pLDDT and pTM-score across both proteins. Mean per-protein pLDDT differences (**c** and **d**) further highlight systematic shifts between predictors in AF2_L_–CF and AF2_L_–AF3 comparisons. Per-residue agreement is evaluated using Bland–Altman (M–P plots **(e–h)**), where differences relative to AF2_L_ are visualized with LOESS smoothing to reveal local biases. Panels **(e–f)** correspond to SGLT1 and **(g–h)** to SGLT2. Confidence ranges follow AlphaFold conventions.

To ensure the generalizability of our findings, we conducted an extensive validation using a cohort of 58 protein alternatives, including 29 SAAVs from the SGLT1 and SGLT2 transporters [15]. As illustrated in Fig. 9a and b, consistent patterns emerge across these proteins: predicted global metrics, such as pTM-score and average pLDDT, diverge significantly between methodologies. Notably, AF3 tends to yield markedly higher confidence scores compared to AF2_L_ and CF, suggesting a more optimistic or refined structural resolution. Conversely, CF consistently produces the lowest pLDDT- and pTM-score values, reinforcing our previous observations. This trend is further supported by the per-residue confidence shifts (${M_x}$, Equation 4) shown in Fig. 9c and d, which highlight the variance between AF2_L_, CF, and AF3.

Furthermore, Fig. 9e–h demonstrate that per-residue confidence deviations are localized within the same structural regions previously identified in the NIS protein.

**Figure 10:**
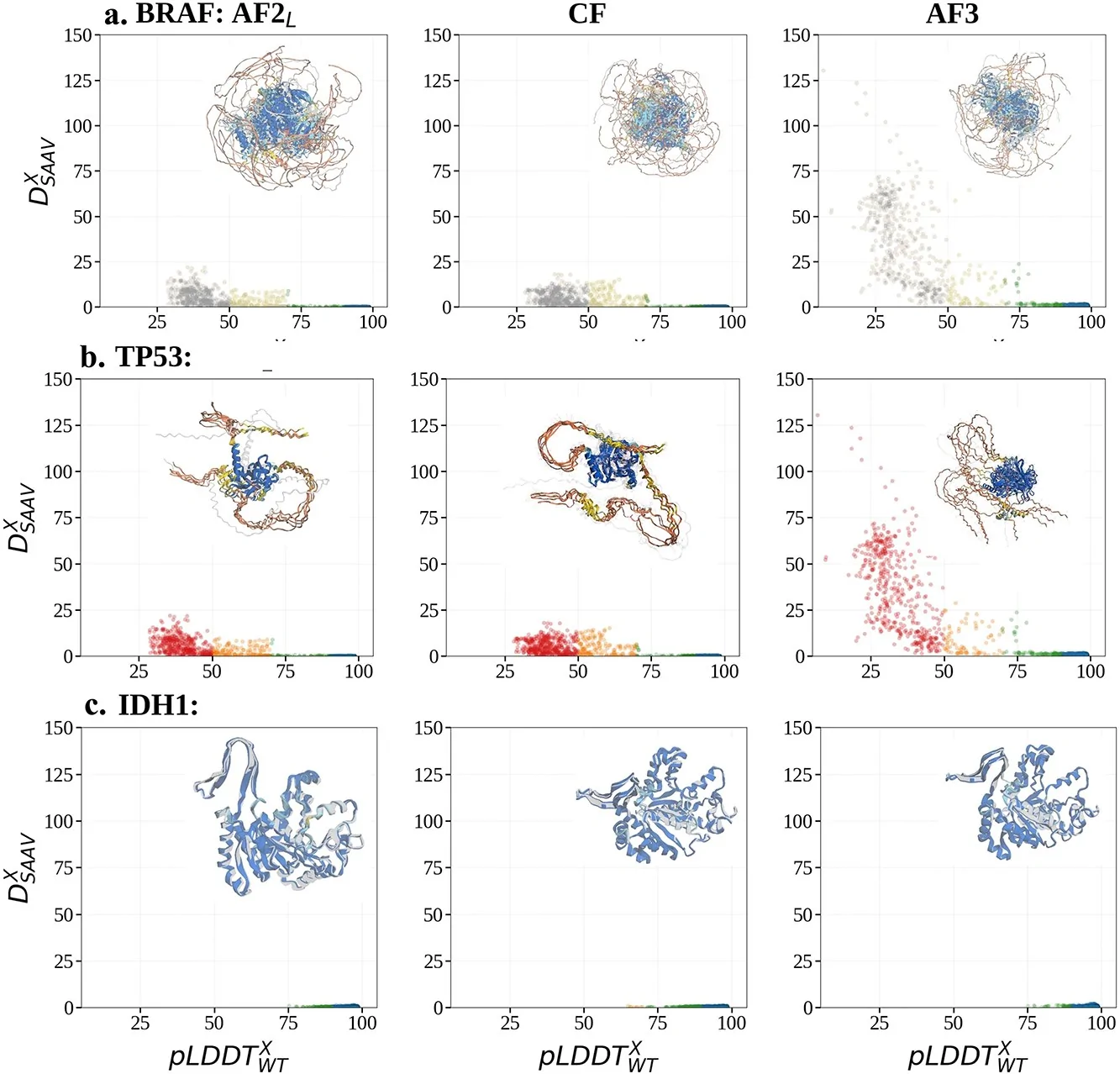
Distance–pLDDT (D–P) plots for BRAF, TP53, and IDH1 variants. Distance–pLDDT (D–P) plots between wild-type (WT) and single amino acid variant (SAAV) models are shown for (a) **BRAF**, (b) **TP53**, and (c) **IDH1**. Distances are evaluated relative to WT pLDDT confidence scores. Columns correspond to prediction methods: AF2_L_ (left), CF (left) and AF3 (right).

To further validate the framework’s sensitivity to SAAVs, we extended our analysis to BRAF, IDH1, and TP53. These proteins were selected due to their critical roles in oncogenesis and the existence of well-characterized clinical mutations that occur in both rigid catalytic domains and flexible regulatory loops, providing a diverse structural landscape for testing in such scenarios. Evaluating a total of 13 mutations (4, 5, and 4 variants, respectively). While the M–P plots (Supplementary Fig. S2) corroborate the baseline patterns of our primary analysis, the D–P plots (Fig. 10) more sharply resolve the structural perturbations introduced by these mutations. Consistent with our initial findings, the most pronounced deviations occur in low-confidence regions, with AF3 again demonstrating the highest degree of coordinate displacement. In contrast, the structural rigidity observed in compact proteins like IDH1—particularly within high-confidence domains—suggests a persistent model-wide stability that resists minor sequence alterations. These confirmatory results underscore the robustness of our benchmarking framework in quantifying the nuanced impact of genetic variants on predicted protein topographies.

## Discussion

The field of computational protein structure prediction continues to advance rapidly [1–5], driven by the emergence of new deep-learning models and alternative implementations designed to broaden applicability and reduce computational requirements [7, 10, 26]. However, systematic comparisons among predictors remain scarce, particularly for novel or unseen proteins not included in model training sets [5, 27]. In such cases, traditional global evaluation metrics often prove insufficient and may obscure important differences in predictor behavior [27].

Our findings illustrate this limitation clearly. For example, although CF has been reported to match or even surpass AF2 performance [10] in benchmarks such as CASP14, such evaluations primarily rely on global summary metrics that can mask meaningful structural discrepancies. Consistent with Edmunds (2024) [27], we observe that global measures such as average pLDDT or pTM-score, while informative, fail to capture local behavior or performance in low-confidence regions. Our extended validation set of seven proteins with diverse architectures further reinforces this conclusion, demonstrating that global averages consistently obscure localized structural divergences regardless of the protein’s fold or complexity.

We show that although these global metrics can reveal broad trends—such as CF’s tendency to produce lower confidence predictions or the generally higher reliability of AF3 uncovers divergence patterns that remain invisible to aggregated measures. Our residue-level profiles demonstrate that while reduced AF2 implementations (e.g. Jackhmmer-based) yield locally distinct structures within a constrained folding manifold, AF3 introduces a paradigm shift through its generative diffusion module. Unlike the physical-spatial constraints of AF2’s Evoformer, AF3’s diffusion process operates by refining atomic coordinates from noise, which often results in biophysically plausible but structurally divergent conformations in low-confidence regions and loops compared to AlphaFold 2-based models [7]. This may suggest that the observed local variance in our AF3 results may be due to how the diffusion model explores the local energy landscape of unstructured segments.

A particularly relevant finding is that reducing computational resources and homology database size does not substantially impact the performance of the evaluated implementations. The strong agreement observed among AF2_L_, AF2_R_, and AF2_F_ provides compelling support for lightweight versions of AlphaFold—including the one executed here on a mid-range NVIDIA 30 series GPU—allowing reliable predictions at a fraction of the computational cost. This outcome directly addresses the methodological challenge raised in the Introduction regarding accessibility and reproducibility across predictors.

Furthermore, we show that residue-level analyses, including Bland–Altman representations (M–P plots) and distance–pLDDT plots (D–P plots), offer a more precise framework for distinguishing predictor behavior. These methods reveal that confidence scores (pLDDT) can be misleading when not interpreted alongside the actual structural congruence. Consistently, we find that divergences between predictors—and between sequence variants—are concentrated in IDRs. This observation agrees with recent insights by Brunello et al. (2025) [28] and Sil et al. (2025) [29], who emphasize that IDPs require specialized analytical strategies, as their dynamic nature cannot be captured by a single folded state.

Finally, 3D visualizations and structural comparisons confirm that both predictor choice and single amino acid variations can induce substantial conformational shifts in flexible regions, even when the ordered core remains highly conserved. This reinforces the importance of integrating analytical tools that jointly consider confidence and local geometry to achieve biologically meaningful interpretations.

Taken together, our findings highlight that reliance on global metrics alone may lead to incomplete conclusions. Residue-level approaches—such as those proposed here—provide a more informative and robust framework for comparing prediction tools in realistic scenarios, particularly when no experimental structure is available.

## Conclusions

The advent of AlphaFold represents a transformative milestone in structural biology—recently acknowledged by the Nobel Prize—and has reshaped the landscape of protein structure prediction. However, as new predictors and alternative implementations emerge, rigorous and context-aware evaluation frameworks are required. Any model proposed as a successor to AlphaFold should achieve comparable or superior performance across established metrics, or deliver clear computational advantages.

Our results show that global measures such as mean pLDDT, TM-score, or RMSD provide an incomplete picture of predictive performance. In contrast, per-residue confidence and coordinate-based analyses expose critical differences among predictors, particularly within intrinsically disordered or flexible regions. These discrepancies caution against overinterpretation of predicted models in mechanistic or functional studies.

Moreover, the analysis of SAAVs reveals that even minimal sequence perturbations can induce detectable conformational changes within low-confidence regions, underscoring their structural sensitivity and the need to evaluate predictive robustness across both models and mutations.

We therefore advocate the integration of complementary analytical and visualization tools—such as M–P and D–P plots and UMAP projections—to enable transparent, reproducible, and biologically meaningful comparisons across structure prediction methods.

**Key points**

- Global structural metrics often mask relevant local differences between protein structure prediction methods.
- Residue-level analyses reveal marked localized divergences, particularly in flexible and intrinsically disordered regions.
- Single amino acid substitutions can induce substantial conformational changes depending on the predictor used.
- Reduced homology databases have minimal impact on predicted structural quality.

## Supplementary Material

Supplementary_Data_bbag461

## Data Availability

The WT human NIS, SGLT1, and SGLT2 protein sequences were obtained from the UniProt database (Uniprot IDs: Q92911, P13866, and P31639, respectively). Benign and pathogenic NIS variants were previously compiled [14]. Pathogenic SGLT1 and SGLT2 variants were manually extracted from the literature [15, 28]. All structural models predicted by the algorithms evaluated in this study, as well as the source data used to generate the figures (including confidence and structural differences matrices), are available at the following GitHub repository: https://github.com/FlorenciaRDiaz/AF_OF_Comparative [22]. Source data for Figs 1–9 and the Supplementary Figure are provided in the same repository.

## Code availability

All analysis scripts (including jupyter notebooks) used for data processing, structural comparisons, and figure generation are available at the following GitHub repository: https://github.com/FlorenciaRDiaz/AF_OF_Comparative [22]. The repository includes documentation and usage instructions to facilitate reproducibility of the analyses.
