# Supplementary material for: Comprehensive evaluation of AlphaFold/OpenFold prediction of experimentally unresolved proteins through novel metrics
Source: Brief Bioinform. 2026 Sep 3;27(5):bbag461. doi: 10.1093/bib/bbag461 (PMC13541284; doi:10.1093/bib/bbag461)

Supplementary Data.

Comprehensive evaluation of AlphaFold/OpenFold prediction of experimentally unresolved proteins through novel metrics

Authors: Florencia R. Díaz[1,3], Daniela Orschanski[3,4], Juan I. Folco[2], Juana Espain Ceci[1], Guadalupe Nibeyro[3,4], Horderlin Robles Vega[5], Juan P. Nicola[6,7], Elmer A. Fernández*[1,3,4,8]

---

1 Facultad de Ciencias Exactas Físicas y Naturales (FCEFyN), Universidad Nacional de Córdoba, Córdoba, Argentina
2 Facultad de Ingeniería, Universidad Católica de Córdoba, Córdoba, Argentina
3 ScireLab, Fundación para el Progreso de la Medicina, Córdoba, Argentina
4 Consejo Nacional de Investigaciones Científicas y Técnicas (CONICET), Córdoba, Argentina
5 Grupo GNOCIX,Facultad de Ciencias e Ingenierías, Universidad del Sinú Elías Bechara Zainum, Montería, Córdoba, Colombia.
6 Departamento de Bioquímica Clínica, Facultad de Ciencias Químicas, Universidad Nacional de Córdoba, Córdoba, Argentina.
7 Centro de Investigaciones en Bioquímica Clínica e Inmunología, Consejo Nacional de Investigaciones Científicas y Técnicas (CIBICI-CONICET), Córdoba, Argentina
8 Universidad Argentina de la Empresa (UADE). Instituto de Tecnología (INTEC). Buenos Aires, Argentina

*** corresponding author**
Elmer A. Fernández, Sicrelab @ Fundación para el Progreso de la Medicina & CONICET, 9 de Julio 941 - X5000EMS - Córdoba, Argentina Córdoba - Argentina. Email: elmer.fernandez@unc.edu.ar

Table of Contents

- **Table S1.** Statistical analysis of global confidence metrics across methods .....**Page 3**
- **Table S2.** Statistical analysis of M distributions for NIS proteins ....**Page 4**
- **Table S3.** Pairwise comparison of per-residue RMSD distributions ....**Page 5**
- **Figure S1.** Structural impact of SAAVs on NIS using D-P plots .... **Page 8**
- **Table S4.** Statistical analysis for SGLT1 and SGLT2 proteins……………………......**Page 9**
- **Figure S2.** M-P plots for BRAF, TP53, and IDH1 variants ....**Page 10**

Table S1. Statistical analysis of global confidence metrics across prediction methods. Pairwise comparisons were performed against the AlphaFold2-Full ($AF2_F$) baseline using the non-parametric Wilcoxon rank-sum test for the extended NIS dataset (*N=181*). *q*-values represent the False Discovery Rate (FDR) correction. Effect sizes are reported as Cohen's *d*. Abbreviations: CF, ColabFold; $AF2_{F/R//L}$, AlphaFold2 Full/Reduced/Local; AF3, AlphaFold3; $OF_{F/R}$, OpenFold Full/Reduced.

Metric	Comparison	p-value	Cohen's	Effect Size	q-value (FDR)
Average pLDDT	AF2F vs AF2R	0,2973	0,096	Small	0,3568
	AF2F vs AF2L	0,9284	0,011	Small	0,9284
	AF2F vs CF	4.00×10−52	2,629	Large	2.40×10−51
	AF2F vs AF3	9.94×10−34	1,496	Large	1.49×10−33
	AF2F vs OFF	4.74×10−49	2,097	Large	1.42×10−48
	AF2F vs OFR	5.12×10−48	2,036	Large	1.02×10−47
pTM Score	AF2F vs AF2R	0,8427	0,018	Small	0,8427
	AF2F vs AF2L	0,5625	0,022	Small	0,6750
	AF2F vs CF	3.92×10−59	2,998	Large	2.35×10−58
	AF2F vs AF3	2.54×10−55	2,841	Large	5.09×10−55
	AF2F vs OFF	1.05×10−55	2,530	Large	3.14×10−55
	AF2F vs OFR	5.26×10−55	2,488	Large	7.88×10−55

Table S2. Statistical analysis of M distributions NIS proteins. Wilcoxon signed-rank tests were performed to evaluate whether the median M ($pLDDT_{AF2_F} - pLDDT_X$)for each predictor differs from zero (baseline defined by $AF2_F$). P-values were adjusted for multiple comparisons using both Bonferroni correction and false discovery rate (FDR). Effect sizes (r) were computed from the standardized test statistic and interpreted as negligible (<0.1), small (0.1–0.3), medium (0.3–0.5), or large (>0.5).

**Algorithm**	**N**	**Wilcoxon W**	**p-value**	**p-bonf**	**q-value (FDR)**	**Effect size (r)**	**Effect magnitude**
AF2L	181	7984	0.722	1.00E+00	7.22E-01	-0.026	Negligible
AF2R	181	7239	0.158	9.48E-01	1.90E-01	-0.105	Small
CF	181	108	1.12E-30	6.71E-30	2.24E-30	-0.856	Large
AF3	181	929	4.14E-25	2.48E-24	6.21E-25	-0.769	Large
OFF	181	34	3.3E-31	1.98E-30	1.40E-30	-0.864	Large
OFR	181	55	4.67E-31	2.80E-30	1.40E-30	-0.861	Large

Table S3. Pairwise statistical comparison of per-residue RMSD distributions between prediction methods across different confidence thresholds. Comparisons were performed using the Wilcoxon signed-rank test with Benjamini–Hochberg correction for multiple testing. Only statistically significant comparisons (FDR-adjusted *p*-value, $q < 0.05$) are shown for clarity. Comparisons may appear repeated across thresholds (ALL, pLDDT ≥ 50, ≥ 70, ≥ 90) as each subset corresponds to a different set of residues defined by the confidence filtering applied to the predicted structures. Therefore, identical method pairs are evaluated independently within each threshold condition.

**Threshold: ALL residues (n = 503)**							
**Method 1**	**Method 2**	**N**	**Wilcoxon W**	**p-value**	**q-value (FDR)**	**Effect size (r)**	**Effect magnitude**
AF2F	AF2R	503	32549	3.31E-21	6.61E-20	-0.42	Medium
AF2L	AF2R	503	35281	7.00E-18	7.00E-17	-0.384	Medium
AF2L	CF	503	40329	1.58E-12	1.05E-11	-0.315	Medium
AF2L	AF3	503	42739	2.48E-10	1.24E-09	-0.282	Small
AF3	OFF	503	43305	7.52E-10	2.51E-09	-0.274	Small
AF2F	AF3	503	47922	2.15E-06	6.14E-06	-0.211	Small
AF2F	CF	503	48275	3.64E-06	9.11E-06	-0.206	Small
AF2F	AF2L	503	49574.5	2.31E-05	5.14E-05	-0.189	Small
CF	OFF	503	53998	4.03E-03	7.32E-03	-0.128	Small
AF2R	OFF	503	55031	1.05E-02	1.61E-02	-0.114	Small
AF2R	AF3	503	55119	1.13E-02	1.62E-02	-0.113	Small
CF	AF3	503	56019	2.40E-02	3.21E-02	-0.101	Small
Threshold: pLDDT≥50							
AF2L	AF2R	500	31621	8.65E-22	1.73E-20	-0.429	Medium
AF2F	AF2R	500	32113	3.74E-21	3.74E-20	-0.422	Medium

AF2L	CF	500	38353	5.95E-14	3.97E-13	-0.336	Medium
AF2L	AF3	500	40762	1.34E-11	6.72E-11	-0.302	Medium
AF3	OFF	500	44464	1.93E-08	6.42E-08	-0.251	Small
AF2F	AF2L	500	44719	3.03E-08	8.66E-08	-0.248	Small
AF2F	AF3	500	47275	2.05E-06	5.11E-06	-0.212	Small
AF2F	CF	500	48222	8.35E-06	1.86E-05	-0.199	Small
CF	AF3	500	54421	1.11E-02	2.23E-02	-0.114	Small
AF2R	AF3	500	54569	1.27E-02	2.31E-02	-0.111	Small
CF	OFF	500	55118	2.02E-02	3.11E-02	-0.104	Small
AF2R	OFF	500	55517	2.79E-02	3.72E-02	-0.098	Negligible
Threshold: pLDDT≥70							
AF2L	AF2R	486	27496	1.53E-24	3.05E-23	-0.464	Medium
AF2F	AF2R	486	29446.5	8.34E-22	8.34E-21	-0.435	Medium
AF2L	AF3	486	31132	1.41E-19	9.40E-19	-0.411	Medium
AF2L	CF	486	32138	2.62E-18	1.31E-17	-0.396	Medium
AF3	OFF	486	35261	1.18E-14	3.92E-14	-0.35	Medium
AF2F	AF3	486	37739	4.56E-12	1.30E-11	-0.314	Medium
AF2F	AF2L	486	41944	2.68E-08	6.70E-08	-0.252	Small
AF2F	CF	486	42699	1.05E-07	2.34E-07	-0.241	Small
CF	AF3	486	42783	1.22E-07	2.44E-07	-0.24	Small
AF2R	AF3	486	43560	4.67E-07	8.49E-07	-0.229	Small
AF2L	OFF	486	52240	2.53E-02	3.89E-02	-0.101	Small
Threshold: pLDDT≥90							
AF2R	AF3	267	7132	1.63E-17	3.27E-16	-0.521	Large
AF2F	AF3	267	7454	1.43E-16	1.43E-15	-0.506	Large

AF2F	OFF	267	8523	1.21E-13	4.03E-13	-0.454	Medium
AF2R	OFF	267	8468	8.69E-14	4.03E-13	-0.457	Medium
AF2L	AF3	267	8734	4.21E-13	1.20E-12	-0.444	Medium
CF	AF3	267	8924	1.26E-12	3.16E-12	-0.434	Medium
AF2L	OFF	267	9383	1.64E-11	3.28E-11	-0.412	Medium
AF2L	AF2R	267	9836	1.82E-10	3.30E-10	-0.39	Medium
CF	OFF	267	9884	2.32E-10	3.58E-10	-0.388	Medium
AF2F	AF2L	267	11548	5.15E-07	7.36E-07	-0.307	Medium
AF2R	CF	267	11816	1.52E-06	2.03E-06	-0.294	Small
AF2F	CF	267	12640	3.24E-05	4.05E-05	-0.254	Small
AF3	OFF	267	13473	4.71E-04	5.24E-04	-0.214	Small
AF2F	AF2R	267	13878	1.49E-03	1.57E-03	-0.194	Small
AF2L	CF	267	15287	3.94E-02	3.94E-02	-0.126	Small

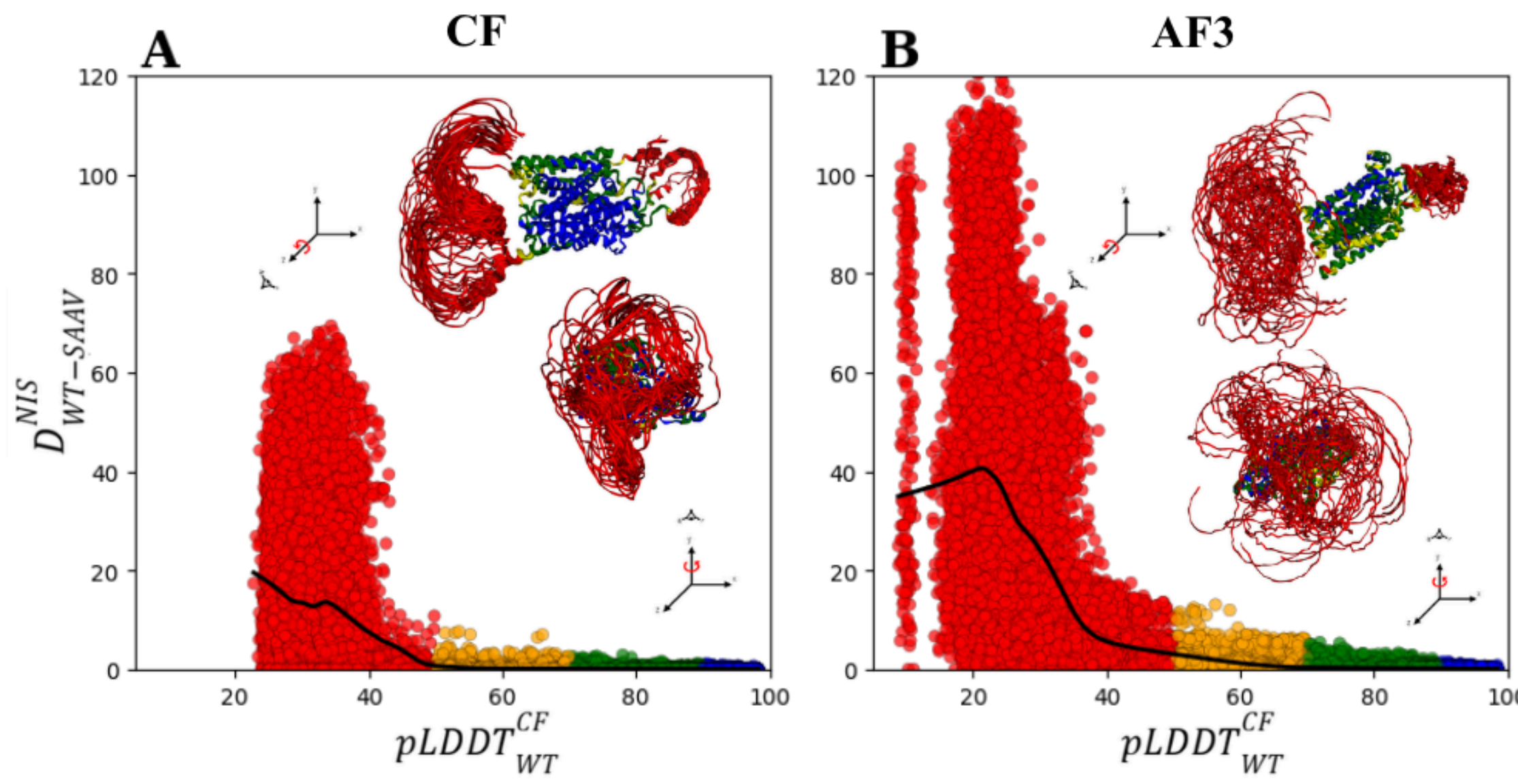

Figure S1. Structural impact of SAAV on NIS. Distance-pLDDT (D-P) plot showing per-residue Cα deviations between Wild-type (WT) and single amino acid variants (SAAVs) NIS models predicted with $AF2_L$. Distances (Y-axis) are plotted against the $AF2_L$ pLDDT confidence scores (X-axis). Points are colored according to AlphaFold2 confidence ranges (very low <50, low <70, confident <90, very high ≥90). The Loess curve indicates that the largest deviations concentrate in low-confidence regions. The inset shows the 3D NIS model colored by pLDDT, highlighting correspondence between high-distance residues and flexible, disordered loops.

Table S4. Statistical analysis for SGLT1 and SGLT2 proteins

	Algorithm	p-value	q-value (FDR)	Effect size (r)	Effect magnitude
SGLT1	AFL-CF_SGLT1	2.00E-06	2.00E-06	883	Large
SGLT1	AFL-AF3_SGLT1	3.73E-09	3.73E-09	1.095	Large
SGLT2	AFL-CF_SGLT2	2.74E-02	2.74E-02	410	Medium
SGLT2	AFL-AF3_SGLT2	6.84E-05	6.84E-05	739	Large

Figure S2. M-P plots for BRAF, TP53, and IDH1 variants. M-P plots showing Bland-Altman analysis between predicted WT and variants counterparts (SAAVs) for (A) BRAF, (B)TP53 and (C)IDH1. Columns represent X: CF (left), column $AF2_L$ (middle) and AF3 (right). (Y-axis) comparing per-residue confidence difference $M^{AF2_L - X}$ to the $AF2_L$ reference pLDDT against Mean per-residue pLDDT differences (X-axis).

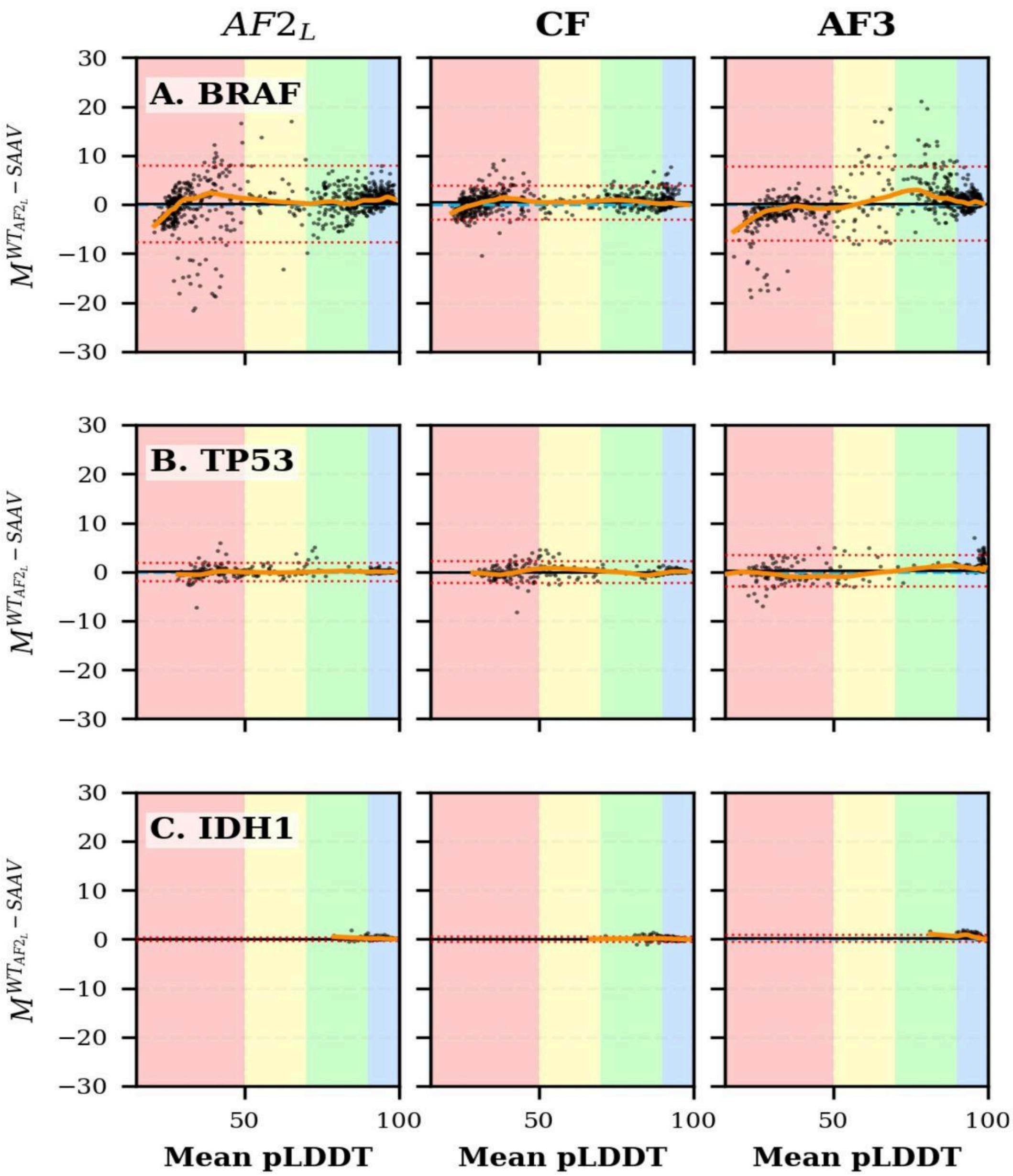

Supplement: Supplementary file 1 [file supplementary_data_bbag461.pdf]
